# DGAT2 reduction and lipid dysregulation drive psoriasis development in keratinocyte-specific SPRY1-deficient mice

**DOI:** 10.1172/jci.insight.192507

**Published:** 2025-07-22

**Authors:** Ying-Ying Li, Li-Ran Ye, Ying-Zhe Cui, Fan Xu, Xi-Bei Chen, Feng-Fei Zhang, Yi Lu, Yu-Xin Zheng, Xiao-Yong Man

**Affiliations:** Department of Dermatology, Second Affiliated Hospital, Zhejiang University School of Medicine, Hangzhou, China.

**Keywords:** Dermatology, Inflammation, Metabolism, Cellular immune response, Lipidomics

## Abstract

Psoriasis is a chronic autoimmune skin disease characterized by abnormal keratinocyte proliferation and immune dysregulation. Altered lipid metabolism has been implicated in its pathogenesis, but the underlying mechanisms remain unclear. In this study, we generated a keratinocyte-specific Sprouty RTK signaling antagonist 1 (SPRY1) knockout (Spry1^ΔEpi^) mouse model, which exhibits psoriasis-like symptoms. Using both psoriasis patient samples and Spry1^ΔEpi^ mice, we investigated the role of diacylglycerol acyltransferase 2 (DGAT2) in psoriasis. Our results show that DGAT2 expression was reduced and glyceride metabolism was disrupted in psoriatic lesions in both patients with psoriasis and Spry1^ΔEpi^ mice. Lipidomic analysis revealed significant alterations in glycerides, glycerophospholipids, sphingolipids, and fatty acids in Spry1^ΔEpi^ mice. At the cellular level, DGAT2 downregulation and lipid dysregulation enhanced TLR3-mediated inflammatory signaling in keratinocytes. Furthermore, increased DGAT2 secretion from keratinocytes promoted CD8^+^ T cell activation, proliferation, and survival, amplifying psoriatic inflammation. These findings highlight the role of DGAT2 and lipid metabolism in the pathogenesis of psoriasis and reveal their interaction with immune responses in psoriasis.

## Abstract 2

The dysregulation of DGAT2 and lipid metabolism enhances TLR3-mediated inflammation in keratinocytes and promotes CD8⁺ T cell activation, driving psoriasis development in keratinocyte-specific SPRY1-deficient mice.

## Graphical abstract

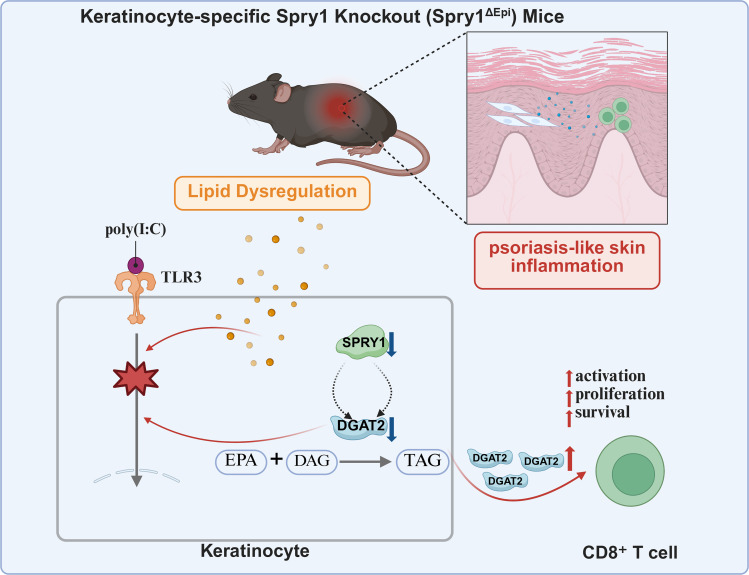

## Introduction

Psoriasis is a common chronic autoimmune skin disorder characterized by excessive immune responses that drive abnormal proliferation and differentiation of epidermal keratinocytes. It presents with erythematous plaques, covered by silvery-white scales, and thickened skin lesions, frequently accompanied by intense pruritus and discomfort (1, 2). The exact cause of psoriasis is unclear, but it is known to be a complex condition influenced by genetic factors, immune system dysfunction, and environmental triggers. Psoriasis is not limited to its dermatological manifestations but is now recognized as a systemic inflammatory disease, often linked to comorbidities such as metabolic syndrome, cardiovascular diseases, inflammatory bowel disease, depression, and metabolic dysfunction–associated steatotic liver disease (MASLD) (3, 4).

Altered lipid metabolism is a key factor in the development of psoriasis. Imbalances in lipid composition are evident not only in the plasma (5) but also in the skin (6–10) and its resident immune cells (11–13). These lipid abnormalities drive inflammation and support keratinocyte hyperproliferation, thereby aggravating the progression of the disease (14, 15). Among the enzymes involved in lipid metabolism, diacylglycerol acyltransferase 2 (DGAT2) plays a key role in lipid metabolism by catalyzing the final step of triglyceride synthesis (16). This enzyme is vital for the formation and maintenance of lipid droplets, which are critical for cellular lipid homeostasis and energy storage (17). DGAT2 dysregulation has been linked to several diseases, including hepatocellular carcinoma (HCC) (18), severe sepsis (19), and MASLD (20), because of its role in regulating lipid homeostasis and cell cycle progression. In the skin, DGAT2 is essential for preserving barrier integrity, as its expression inversely correlates with transepidermal water loss, a phenomenon notably observed in conditions such as atopic dermatitis (21).

SPRY1 (Sprouty RTK signaling antagonist 1), a member of the Sprouty protein family, functions as a key negative regulator of receptor tyrosine kinase (RTK) signaling (22). It plays a particularly important role in modulating the MAPK/ERK pathway, a critical cascade involved in regulating cell proliferation, differentiation, and survival (23). Our previous studies have shown that SPRY1 possesses antiinflammatory properties and plays a protective role in psoriasis by regulating cutaneous innate immune responses (24). Notably, SPRY1 expression is reduced in lesional psoriatic skin (25). To investigate its role further, we generated a keratinocyte-specific Spry1-knockout (Spry1^ΔEpi^) mouse model (26), which exhibited psoriasis-like features, including scaling, skin thickening, and hyperpigmentation.

In this study, we investigated the underlying causes of psoriasis-like symptoms in Spry1^ΔEpi^ mice. We observed decreased DGAT2 expression and abnormal glyceride metabolism in the epidermis of both patients with psoriasis and Spry1^ΔEpi^ mice. The disruption of DGAT2 and lipid metabolism led to immune dysfunction in keratinocytes and CD8^+^ T cells, further promoting inflammatory responses.

## Results

### Dysregulated glyceride metabolism and decreased DGAT2 expression in lesional psoriatic skin.

**Figure 1:**
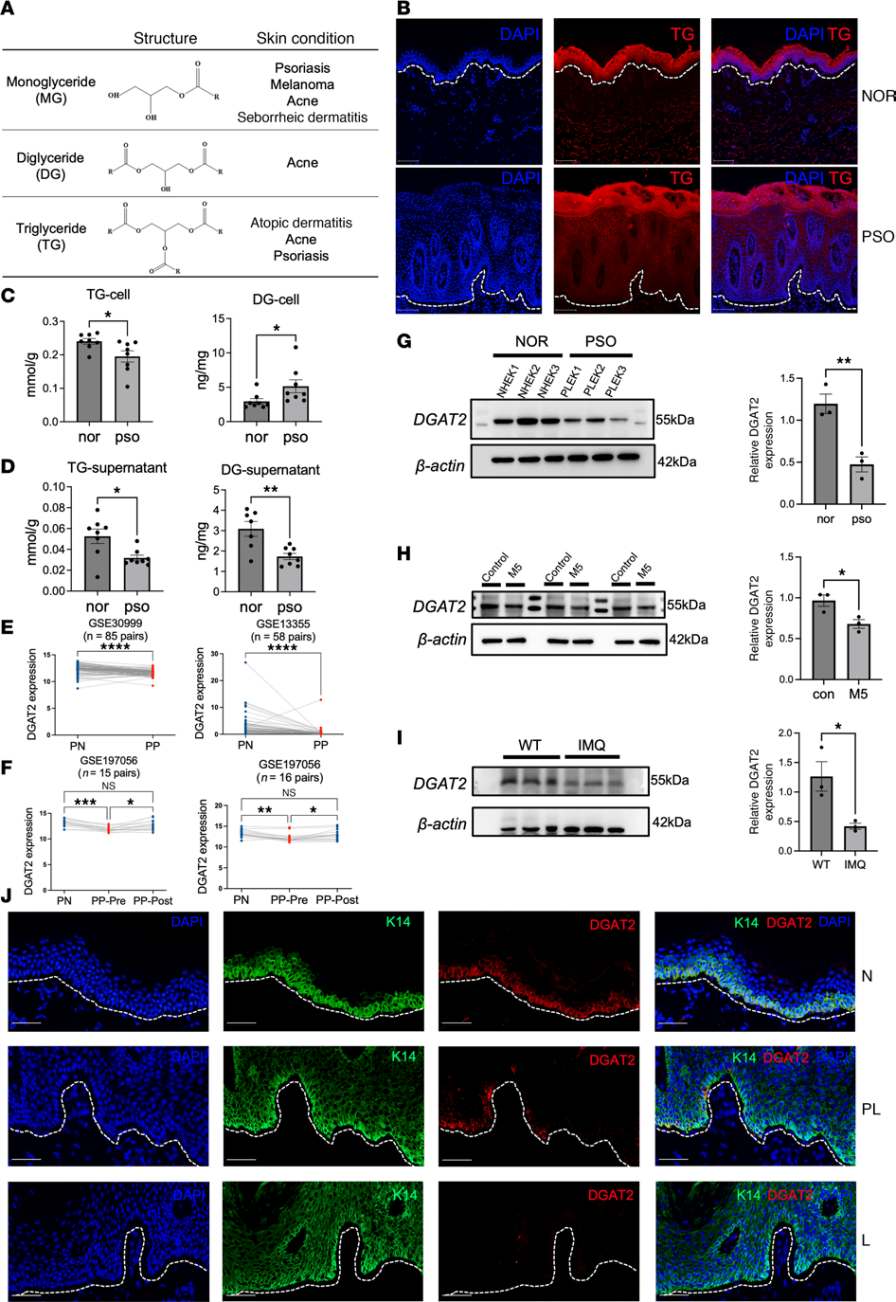
Dysregulated glyceride metabolism and decreased DGAT2 expression in lesional psoriatic skin. (**A**) Summary table linking different glycerides with various skin conditions. (**B**) Triglyceride (TG) staining images showing epidermal TG distribution in patients with psoriasis (PSO) compared with healthy controls (NOR). (**C** and **D**) ELISA quantification of TG and DG levels in keratinocytes and supernatants from NOR and PSO samples. *n* = 8 samples per group. (**E**) Analysis of DGAT2 mRNA expression in nonlesional (PN) and psoriatic lesional (PP) skin using GEO datasets GSE30999 and GSE13355. (**F**) GSE197056 dataset analysis showing restoration of DGAT2 expression in lesional skin following treatment with secukinumab (anti–IL-17A) or guselkumab (anti–IL-23). (**G**) Western blot analysis showing reduced DGAT2 expression in keratinocytes from PSO compared with NOR. (**H**) In vitro stimulation of normal keratinocytes with the M5 cytokine cocktail resulting in decreased DGAT2 expression. (**I**) DGAT2 expression decreased in an IMQ-induced psoriasis-like mouse model compared with wild-type (WT) controls. (**J**) Immunofluorescence staining of skin samples showing progressive reduction in DGAT2 expression from healthy epidermis (N) to peri-lesional (PL) and psoriatic lesional (L) skin. For all images, the original magnification is 20×. Scale bars = 50 μm. For Western blotting, 20 μg of protein is loaded per well. *n* = 3 samples per group. Two-tailed Student’s *t* test was performed. *P < 0.05, **P < 0.01, ***P < 0.001, ****P < 0.0001.

The table (Figure 1A) summarizes the link between glycerides — e.g., triglyceride (TG), diglyceride (DG), monoglyceride (MG) — and various skin conditions, including psoriasis, atopic dermatitis, acne, seborrheic dermatitis, and skin cancer (27–32). This highlights how glyceride metabolism may contribute to the development of these disorders. We examined TG levels in human psoriatic and normal skin using TG staining (Figure 1B). The results showed that TG was abundant in the stratum corneum of lesional psoriatic skin but greatly reduced in the stratum lucidum, granulosum, and spinosum compared with normal skin. To further verify these findings, we evaluated TG levels in keratinocytes isolated from patients with psoriasis and healthy individuals. TG staining showed a substantial reduction in TG levels in keratinocytes from patients compared with healthy controls (Supplemental Figure 1; supplemental material available online with this article; https://doi.org/10.1172/jci.insight.192507DS1). To assess intracellular and extracellular TG and DG levels in keratinocytes, we performed ELISA. Psoriatic keratinocytes exhibited significantly lower intracellular TG levels and markedly elevated DG levels compared with normal keratinocytes (Figure 1C). In the culture supernatants, both TG and DG levels were reduced (Figure 1D).

Diacylglycerol acyltransferase 2 (DGAT2) is a key enzyme in the synthesis of TG from DG and fatty acids (FAs). This process represents the final step in the TG synthesis pathway and is a crucial step in fat synthesis and storage. Given the abnormalities of glycerides in psoriasis lesions and the key role of DGAT2 in glyceride metabolism, we examined the expression of DGAT2 in psoriasis using datasets from the National Center for Biotechnology Information (NCBI) Gene Expression Omnibus (GEO), such as GSE30999 and GSE13355. The analysis revealed that DGAT2 mRNA expression was significantly decreased in psoriatic lesional skin compared with nonlesional skin (Figure 1E). This finding was verified by the GSE197056 dataset, which demonstrated a similar reduction in DGAT2 expression in psoriatic lesions. Interestingly, treatment with secukinumab (anti–IL-17A monoclonal antibody) or guselkumab (anti–IL-23 monoclonal antibody) restored DGAT2 expression levels to those comparable to nonlesional skin (Figure 1F).

To validate these findings at the protein level, Western blot (WB) analysis was performed using keratinocytes from both healthy individuals and patients with psoriasis. The results showed a notable reduction in DGAT2 protein levels in patients compared with healthy controls (Figure 1G). Furthermore, in an in vitro psoriasis model where normal keratinocytes were stimulated with the M5 cytokine cocktail (TNF-α, IL-17A, IL-22, IL-1α, and oncostatin M), DGAT2 expression was similarly reduced (Figure 1H). Consistent with these observations, DGAT2 protein levels were also decreased in the epidermis of the imiquimod-induced (IMQ-induced) psoriasis-like mouse model (Figure 1I). Quantitative analysis of WB data confirmed these differences, with statistically significant results observed across all replicates. To further assess the spatial distribution and expression levels of DGAT2, immunofluorescence staining was performed on skin samples from healthy individuals and patients with psoriasis (peri-lesional and lesional regions). The results demonstrated a progressive decline in DGAT2 expression, with the highest levels in healthy epidermis, reduced levels in peri-lesional areas, and the lowest levels in psoriatic lesions (Figure 1J). Notably, DGAT2 was primarily localized in the basal layer of the epidermis.

### Spry1^ΔEpi^ mice exhibit psoriasis-like symptoms and dysregulated glyceride metabolism.

**Figure 2:**
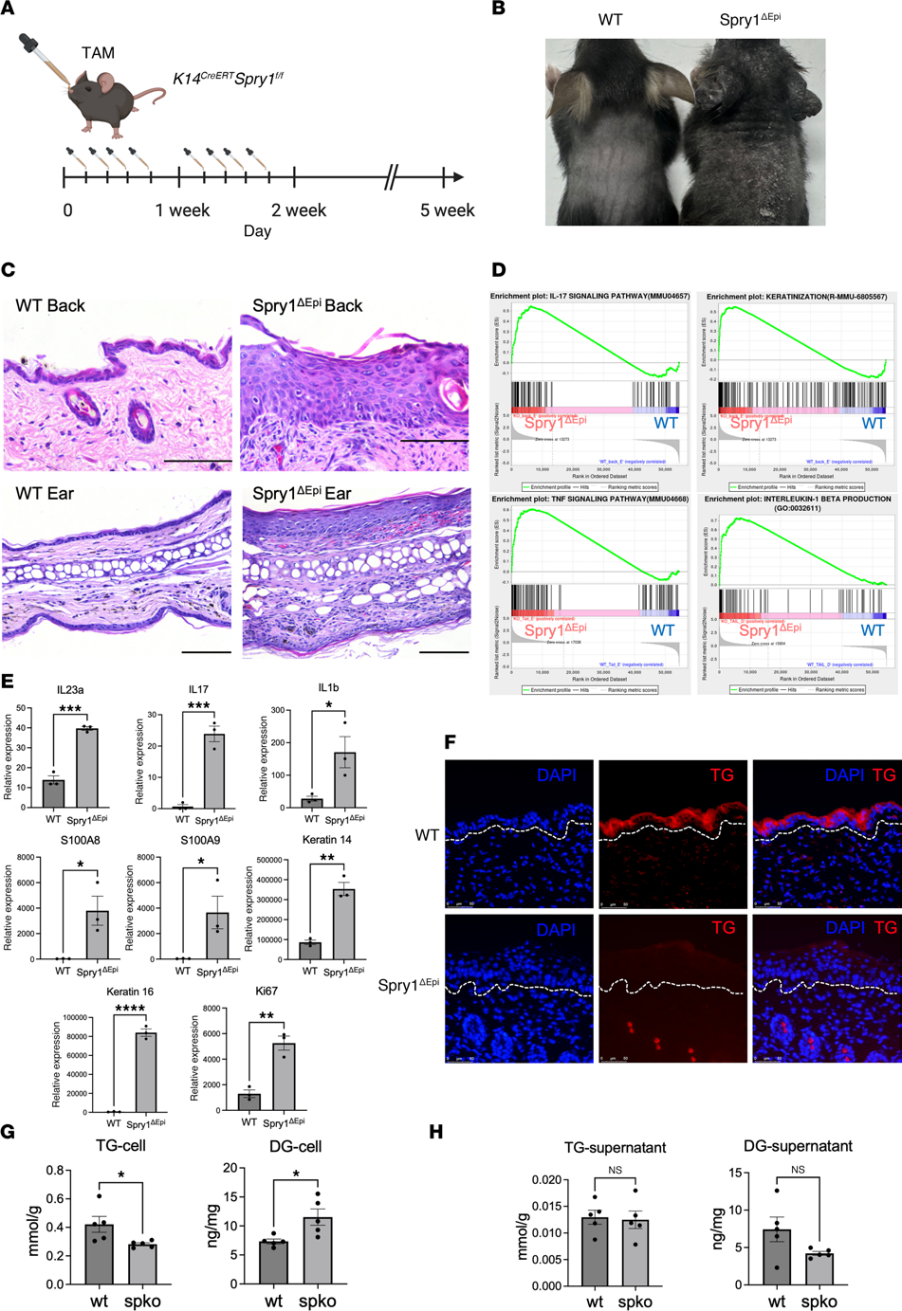
Spry1^ΔEpi^ mice exhibit psoriasis-like symptoms and dysregulated glyceride metabolism. (**A**) Experimental timeline of tamoxifen (TAM) administration to establish K14^CreERT^ Spry1^fl/fl^ (Spry1^ΔEpi^) mice. (**B**) Images of Spry1^ΔEpi^ and WT mice showing psoriasis-like skin phenotypes in Spry1^ΔEpi^ mice. (**C**) Hematoxylin and eosin (HE) staining of the back and ear skin of WT and Spry1^ΔEpi^ mice. (**D**) Gene set enrichment analysis (GSEA) showing significant upregulation of the IL-17 signaling pathway, TNF signaling pathway, IL-1 beta production, and keratinization in the skin of Spry1^ΔEpi^ mice. (**E**) Increased expression of inflammatory cytokines (IL23a, IL17, IL1b, S100A8, S100A9), keratinization (Keratin14, Keratin16), and cell proliferation (Ki67) in the epidermis of Spry1^ΔEpi^ mice. *n* = 3 mice per genotype. (**F**) TG staining images showing epidermal TG distribution in Spry1^ΔEpi^ compared with WT mice. (**G** and **H**) ELISA quantification of TG and DG levels in keratinocytes and supernatants from Spry1^ΔEpi^ and WT mice. *n* = 5 samples per group. For all images, the original magnification is 20×. Scale bars = 50 μm. Two-tailed Student’s *t* test was performed. *P < 0.05, **P < 0.01, ***P < 0.001, ****P < 0.0001.

Based on our previous findings that Spry1 in keratinocytes suppresses psoriasis-like skin inflammation by inhibiting cathelicidin production and cutaneous immune activation, we next examined the impact of Spry1 loss in epidermal keratinocytes (25). We generated Spry1^ΔEpi^ mice by crossing Spry1-floxed mice with K14-Cre-ERT2 mice as previously described (26, 33). Spry1^ΔEpi^ mice were established by administering tamoxifen via oral gavage 4 times per week for 5 weeks (Figure 2A). These mice exhibited psoriasis-like phenotypes, including scaling, epidermal thickening, and hyperpigmentation (Figure 2B). Hematoxylin and eosin (HE) staining of the back and ear skin of WT and Spry1^ΔEpi^ mice revealed epidermal thickening and infiltration of inflammatory cells in Spry1^ΔEpi^ mice (Figure 2C). Next, we performed RNA sequencing to compare skin samples obtained from Spry1^ΔEpi^ mice and WT mice after 5 weeks of tamoxifen induction. Gene set enrichment analysis (GSEA) showed significant upregulation of the IL-17 signaling pathway, TNF signaling pathway, IL-1 beta production, and keratinization in the skin of Spry1^ΔEpi^ mice (Figure 2D). Furthermore, the expression of inflammatory cytokines (IL23a, IL17, IL1b, S100A8, S100A9), keratinization (Keratin14, Keratin16), and cell proliferation (Ki67) were significantly upregulated in the epidermis of Spry1^ΔEpi^ mice (Figure 2E).

Similar to the glyceride abnormalities observed in psoriasis lesions, in the IMQ-induced psoriasis-like mouse model (data derived from ref. 6), TG levels gradually decreased and DG levels increased in skin samples as the IMQ induction time was extended (Supplemental Figure 2A). Similarly, TG staining of skin from Spry1^ΔEpi^ and WT mice revealed a substantial reduction in TG levels in the epidermis of Spry1^ΔEpi^ mice (Figure 2F). These Spry1^ΔEpi^ mice exhibited epidermal thickening and almost complete loss of TG staining. To further verify these findings, we evaluated TG levels in keratinocytes isolated from Spry1^ΔEpi^ mice and WT controls. Keratinocytes from Spry1^ΔEpi^ mice showed significantly lower TG levels compared with those from WT mice (Supplemental Figure 2B). Next, we performed ELISA to measure intracellular and extracellular TG and DG levels in keratinocytes. Similarly, intracellular TG levels were significantly lower in Spry1^ΔEpi^ keratinocytes than in WT controls, while DG levels were elevated (Figure 2G). However, no significant differences in extracellular TG and DG levels were observed between Spry1^ΔEpi^ and WT keratinocytes (Figure 2H).

### SPRY1 is associated with DGAT2 expression and stability in keratinocytes.

**Figure 3:**
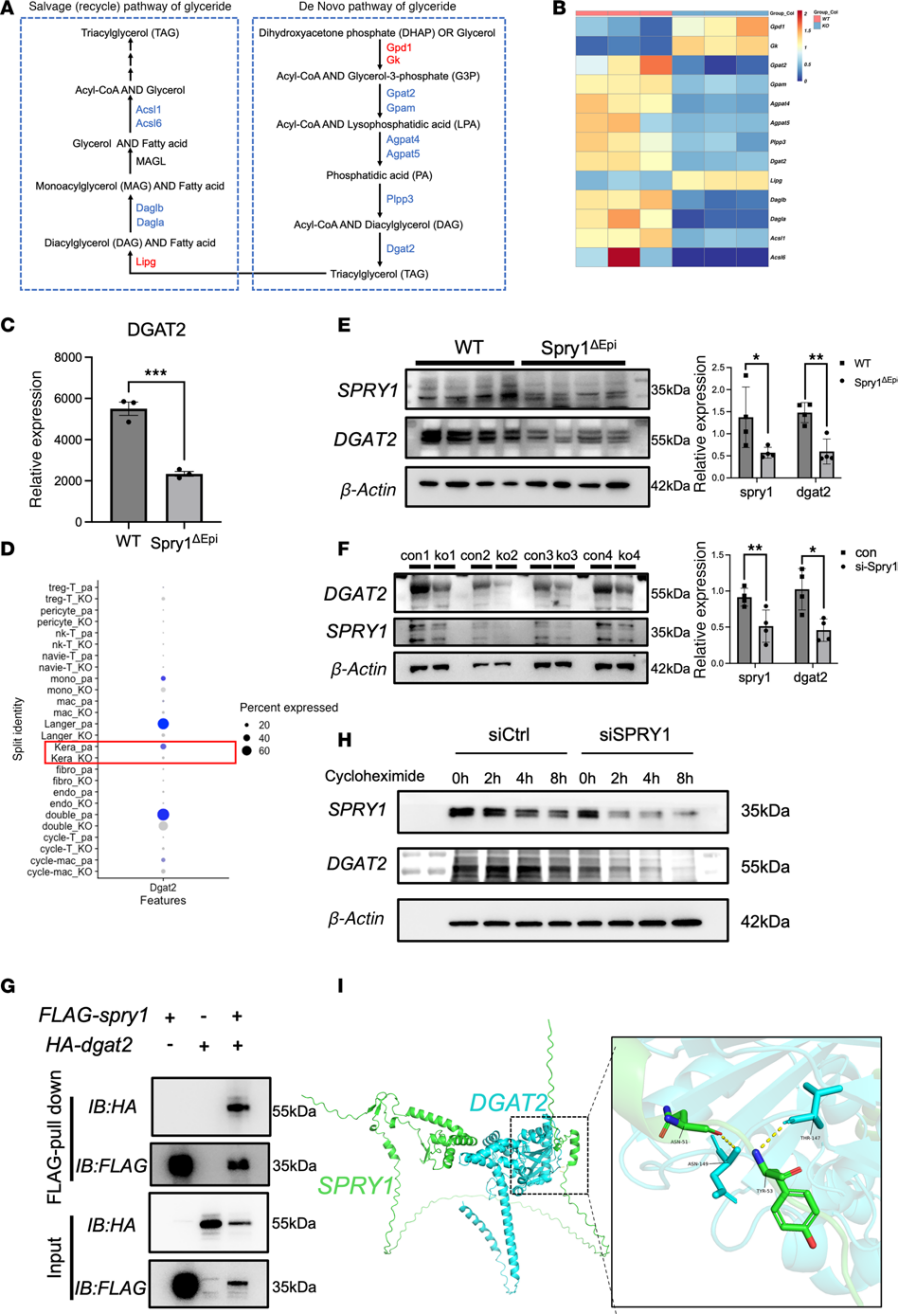
SPRY1 is associated with DGAT2 expression and stability in keratinocytes. (**A**) Two main pathways in glyceride metabolism. (**B**) Heatmap showing differential expression of glyceride metabolism–related genes in Spry1^ΔEpi^ and WT mice. (**C**) RNA sequencing analysis verifying reduced DGAT2 expression in epidermis of Spry1^ΔEpi^ compared with WT mice. *n* = 3 mice per genotype. (**D**) Single-cell RNA sequencing showing DGAT2 expression across various cell populations in Spry1^ΔEpi^ mice. (**E**) Western blot showing decreased DGAT2 expression in epidermis from Spry1^ΔEpi^ compared with WT mice. (**F**) Western blot analysis showing reduced DGAT2 expression following SPRY1 deletion in keratinocytes. (**G**) Co-immunoprecipitation assay demonstrating a direct interaction between SPRY1 and DGAT2 in mouse keratinocytes. (**H**) Western blot analysis showing accelerated degradation of DGAT2 protein upon SPRY1 knockdown, assessed over time following cycloheximide treatment to inhibit new protein synthesis. (**I**) Molecular modeling using AlphaFold3 predicts a stable interaction interface between SPRY1 (green) and DGAT2 (blue), with hydrogen bonds between specific residues of the 2 proteins. The magnified view highlights hydrogen bonds supporting the interaction. For Western blotting, 20 μg of protein is loaded per well. *n* = 4 samples per group. Two-tailed Student’s *t* test was performed. *P < 0.05, **P < 0.01, ***P < 0.001.

Glyceride metabolism consists of 2 main pathways: the glyceride salvage pathway and the de novo glyceride synthesis pathway (Figure 3A). A heatmap displayed the expression of enzymes involved in these glyceride metabolic pathways in the epidermis of Spry1^ΔEpi^ mice compared with WT controls (Figure 3B). DGAT2, a key enzyme in the conversion of DG to TG, showed a significant reduction in the epidermis of Spry1^ΔEpi^ mice (Figure 3C). This finding mirrors the decreased DGAT2 expression observed in psoriatic lesions. Correlation analysis using GEO datasets (GSE13355 and GSE30999) revealed a significant positive correlation between SPRY1 and DGAT2 expression in psoriatic skin (Supplemental Figure 3A). Single-cell RNA sequencing of skin samples from Spry1^ΔEpi^ and WT mice further verified the decreased expression of DGAT2 in Langerhans cells, keratinocytes, and double-positive cell populations in Spry1^ΔEpi^ mice (Figure 3D). Double-positive cells were identified during our clustering analysis as expressing both the Langerhans cell marker and the macrophage marker, indicating a mixed phenotype not previously characterized to our knowledge. WB analysis of epidermal protein extracts from Spry1^ΔEpi^ and WT mice also verified a substantial reduction in DGAT2 protein levels in Spry1^ΔEpi^ mice (Figure 3E). Quantification of the protein expression levels showed statistically significant differences, validating the downregulation of DGAT2 in the absence of SPRY1. To determine whether SPRY1 directly regulates DGAT2 expression in keratinocytes, we cultured keratinocytes from Spry1^ΔEpi^ mice and induced SPRY1 deletion via tamoxifen treatment for 3 days. WB analysis revealed that SPRY1 deletion significantly reduced DGAT2 expression in these keratinocytes (Figure 3F). Protein quantification further confirmed these findings.

To investigate the regulatory mechanism underlying DGAT2 downregulation, we first asked whether SPRY1 controls *Dgat2* transcription. Chromatin immunoprecipitation (ChIP) assays in mouse keratinocytes showed that SPRY1 does not bind to the *Dgat2* promoter (Supplemental Figure 3B), suggesting that SPRY1 does not regulate *Dgat2* transcription through direct promoter interaction. We also overexpressed SPRY1 in primary keratinocytes and observed no increase in DGAT2 protein levels (Supplemental Figure 3C), further indicating that transcriptional regulation is unlikely.

Given the observed protein-level correlation between SPRY1 and DGAT2, we next explored whether SPRY1 modulates DGAT2 posttranslationally. Co-immunoprecipitation experiments verified a direct interaction between SPRY1 and DGAT2 in keratinocytes (Figure 3G), and immunofluorescence staining demonstrated colocalization of both proteins (Supplemental Figure 3D). To test whether SPRY1 affects DGAT2 protein stability, we treated keratinocytes with cycloheximide to block new protein synthesis and monitored DGAT2 degradation over time. DGAT2 degraded more rapidly in cells lacking SPRY1, indicating that SPRY1 may stabilize DGAT2 protein by protecting it from degradation (Figure 3H).

To further support the physical interaction, molecular modeling using AlphaFold3 predicted a stable complex between SPRY1 and DGAT2, with hydrogen bonds forming between SPRY1 (residues TYR-53, ASN-51, and THR-37) and DGAT2 (residues THR-147, ASN-149, and ARG-154). All predicted hydrogen bonds were within a reasonable spatial distance (<3.6 Å), supporting the formation of a stable interaction interface (Supplemental Figure 3E). The overall structure (Figure 3I) illustrates the interaction between SPRY1 (green) and DGAT2 (blue). A magnified view (Figure 3I) highlights the specific hydrogen bonds at the binding interface. Notably, due to visualization software thresholds, only hydrogen bonds <3.50 Å are displayed in the magnified image. Together, these findings suggest that SPRY1 may not regulate DGAT2 at the transcriptional level but could influence DGAT2 protein stability through interaction. The observed positive correlation between SPRY1 and DGAT2 mRNA levels in psoriasis datasets may reflect a shared upstream regulatory mechanism, which needs further investigation.

### Altered lipid profiles in the epidermis and plasma of Spry1^ΔEpi^ mice.

**Figure 4:**
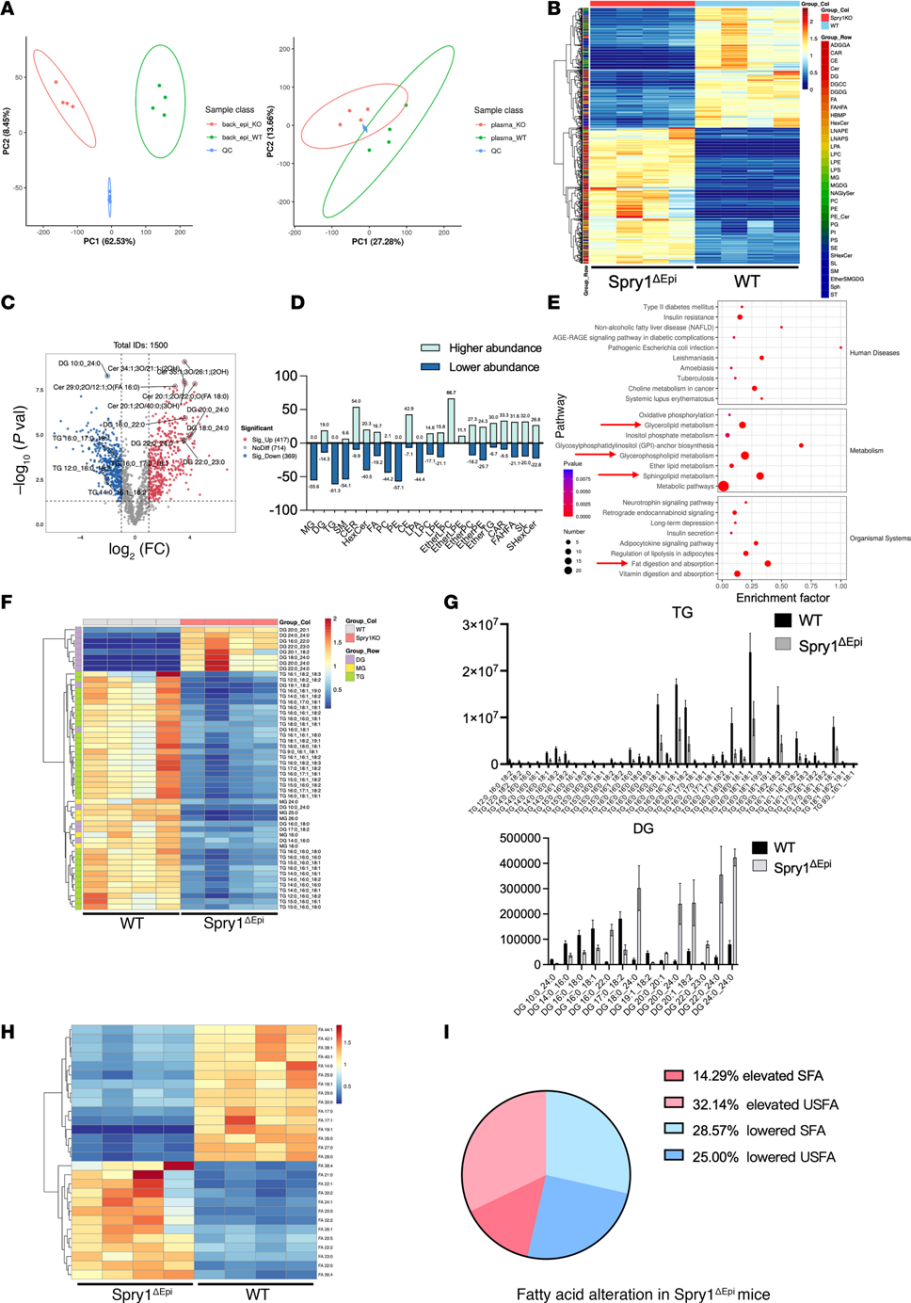
Altered lipid profiles in the epidermis and plasma of Spry1^ΔEpi^ mice. (**A**) PCA showing distinct clustering of lipidomic profiles in the epidermis and plasma between Spry1^ΔEpi^ and WT mice. (**B**) Heatmap highlighting altered lipid classes, including DG, TG, ceramides, phospholipids, and lysophospholipids, in Spry1^ΔEpi^ epidermis. (**C**) Volcano plot identifying 417 significantly upregulated and 369 significantly downregulated lipid species in Spry1^ΔEpi^ epidermis. (**D**) Proportional analysis verifying widespread lipid alterations in Spry1^ΔEpi^ epidermis. (**E**) KEGG pathway enrichment analysis that identifies disruptions in glyceride, glycerophospholipid, and sphingolipid metabolism in Spry1^ΔEpi^ epidermis. (**F**) Heatmap of MG, DG, and TG showing notable differences in Spry1^ΔEpi^ epidermis compared with WT. (**G**) Quantitative analysis revealing TG reduction and DG elevation in Spry1^ΔEpi^ mice. (**H** and **I**) FA profiling that demonstrates altered proportions of saturated and unsaturated fatty acids in Spry1^ΔEpi^ mice. For lipid profiling, *n* = 4 mice per genotype.

To further investigate the lipid metabolic dysregulation in the Spry1^ΔEpi^ mice, lipidomic profiling was performed on epidermal and plasma samples from Spry1^ΔEpi^ and WT mice. Principal component analysis (PCA) revealed a clear separation between Spry1^ΔEpi^ and WT samples in epidermal lipid profiles, indicating a substantial effect of SPRY1 deletion on lipid metabolism in the epidermis. A similar but less pronounced clustering pattern was observed in plasma lipid profiles, suggesting that the metabolic disruption was more localized to the epidermis. Tight clustering of quality control (QC) samples confirmed the reliability and reproducibility of the analysis (Figure 4A). Heatmap clustering analysis further highlighted distinct lipidomic profiles between Spry1^ΔEpi^ and WT groups. Specific lipid classes, including glycerides, fatty acids, ceramides, sphingolipids, phospholipids, and lysophospholipids, were significantly altered in the epidermis of Spry1^ΔEpi^ mice, suggesting systemic disruption of lipid homeostasis (Figure 4B). Volcano plot analysis identified 1,500 lipid species, of which 417 were significantly upregulated and 369 were significantly downregulated in Spry1^ΔEpi^ mice compared with WT mice (Figure 4C). Proportional analysis of altered lipid species further verified marked dysregulation in Spry1^ΔEpi^ epidermis (Figure 4D). Pathway enrichment analysis using Kyoto Encyclopedia of Genes and Genomes (KEGG) databases identified multiple significantly altered metabolic pathways, including glyceride metabolism, glycerophospholipid metabolism, and sphingolipid metabolism (Figure 4E). Other pathways, such as fat digestion and absorption, also showed dysregulation, emphasizing broader metabolic consequences of SPRY1 deletion. Quantitative analysis revealed a marked reduction in TG levels and an increase in DG abundance in Spry1^ΔEpi^ mice compared with WT mice. Among the 62 TG and 42 DG species detected, 38 TGs and 14 DGs showed significant differences between groups. In contrast, MG levels displayed minimal changes. Only 9 MG species were identified in total, of which 5 exhibited significant changes (Figure 4, F and G; see row annotations in Figure 4F). Additionally, the proportion of unsaturated fatty acids (UFAs) increased significantly in Spry1^ΔEpi^ mice, including notable elevations in specific species, such as eicosapentaenoic acid (EPA) (Figure 4, H and I).

Lipid alterations in the plasma of Spry1^ΔEpi^ mice were observed but were less pronounced compared with the epidermis. These changes primarily involved phospholipids, lysophospholipids, and glycerides (Supplemental Figure 4A). Pathway enrichment analysis of plasma samples revealed trends similar to those in the epidermis, with notable disruptions in glycerides and sphingolipid metabolism (Supplemental Figure 4B). However, alterations in DG and TG species within plasma were less substantial compared with epidermis (Supplemental Figure 4, C and D). These results demonstrate that SPRY1 deletion in Spry1^ΔEpi^ mice leads to crucial changes in lipid metabolism, especially in the epidermis. Key lipid species are notably altered, which may contribute to the psoriasis-like symptoms in Spry1^ΔEpi^ mice.

### DGAT2 inhibition and EPA amplify TLR3-mediated inflammatory responses in keratinocytes.

**Figure 5:**
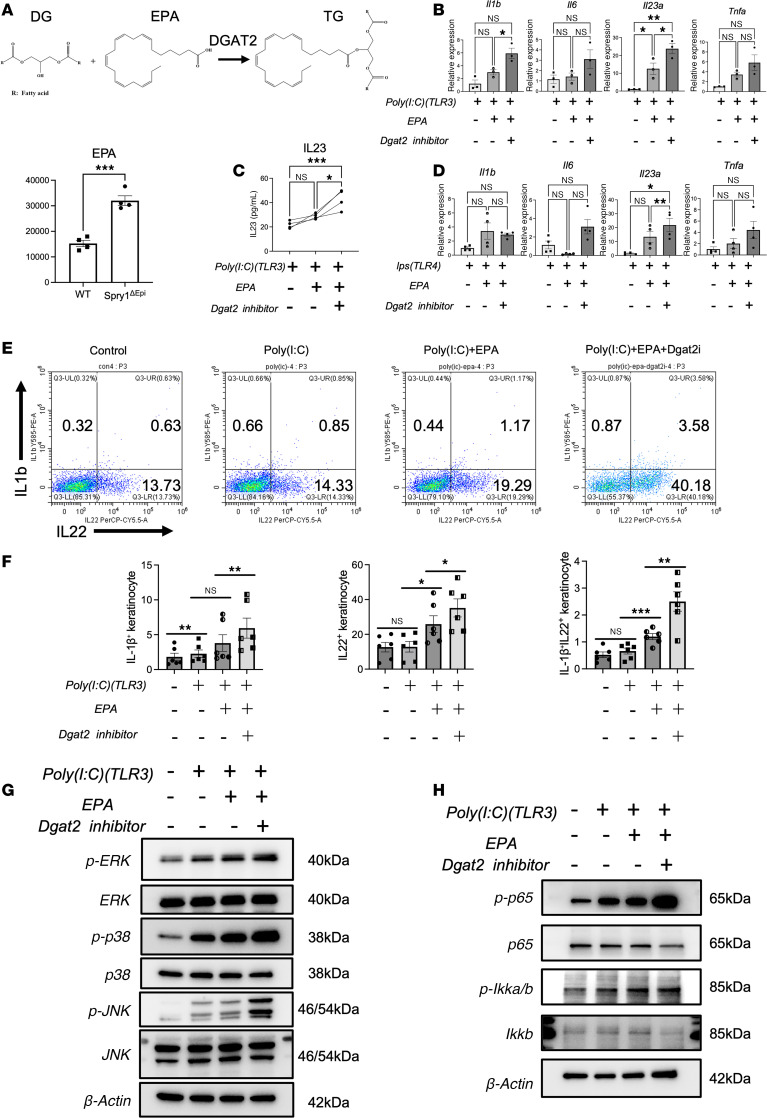
DGAT2 inhibition and EPA amplify TLR3-mediated inflammatory responses in keratinocytes. (**A**) DGAT2 catalyzes TG synthesis from DG and FAs, like EPA. Spry1^ΔEpi^ mouse epidermis shows significantly higher EPA levels. *n* = 4 mice per genotype. (**B**) Quantitative reverse transcription (qRT-PCR) analysis of cytokine expression (IL-1β, IL-6, IL-23, and TNF-α) in keratinocytes treated with poly(I:C), EPA, and DGAT2 inhibitor (DGAT2i). *n* = 3 samples per group. (**C**) ELISA quantification of IL-23 in keratinocyte supernatants showing increased secretion with EPA and DGAT2i. *n* = 4 samples per group. (**D**) qRT-PCR analysis of cytokine expression in keratinocytes treated with LPS, EPA, and DGAT2i. *n* = 4 samples per group. (**E** and **F**) Flow cytometry analysis showing increased proportions of IL-1β^+^IL-22^+^ keratinocytes following EPA and DGAT2i treatment. *n* = 6 samples per group. (**G**) Western blot analysis of MAPK pathway activation that shows increased phosphorylation of ERK, p38, and JNK with poly(I:C), EPA, and DGAT2i. (**H**) Western blot analysis of NF-κB pathway activation that shows enhanced phosphorylation of p65 and IκBα with poly(I:C), EPA, and DGAT2i. For Western blotting, 20 μg of protein is loaded per well. *n* = 3 samples per group. Repeated measures 1-way ANOVA was performed. *P < 0.05, **P < 0.01, ***P < 0.001.

DGAT2 could catalyze the conversion of DG and EPA (a UFA) into TG, and its expression is reduced in keratinocytes of Spry1^ΔEpi^ mice. Lipid metabolism was disrupted in the epidermis of Spry1^ΔEpi^ mice, including an increase in EPA (Figure 5A). These findings suggest that the reduced DGAT2 expression and lipid metabolism disruption may contribute to inflammatory responses associated with psoriasis. To further explore this, we investigated the interplay between lipid dysregulation and immune activation in keratinocytes. In our system, EPA or DGAT2 inhibition alone did not markedly activate keratinocytes, suggesting that lipid dysregulation acts as an amplifier rather than an initiator of inflammation. Previous studies have shown that TLRs play critical roles in the innate immune system by recognizing various lipids, including FAs (34–36). Inhibition of signaling downstream of TLRs could reverse the pathological molecular changes observed in human psoriatic lesions (37). Keratinocytes express functional TLR3 and -4, and stimulation of these cells with TLR3 and TLR4 ligands induces immune-related responses (38). Therefore, we treated keratinocytes with the TLR3 agonist poly(I:C) and subsequently added EPA and the DGAT2 inhibitor (DGAT2i). The addition of EPA to poly(I:C)-stimulated keratinocytes led to a notable increase in IL-23 expression, while IL-1β, IL-6, and TNF-α levels showed an upward trend but did not reach statistical significance (Figure 5B). Further addition of DGAT2i significantly amplified IL-23 expression, while the changes in other cytokines remained nonsignificant. ELISAs verified these observations, demonstrating that IL-23 secretion in keratinocyte culture supernatants was moderately increased by EPA but significantly elevated by the addition of DGAT2i (Figure 5C). Similar trends were observed in keratinocytes stimulated with TLR4 agonist LPS, though the effects were less pronounced compared with poly(I:C) (Figure 5D).

IL-1β^+^ and IL-22^+^ keratinocytes can be detected in human epidermis (Supplemental Figure 5B), indicating that keratinocytes are capable of producing these cytokines under physiological conditions. Flow cytometry analysis of keratinocytes revealed that poly(I:C) stimulation alone had an impact on IL-1β production in keratinocytes. Additionally, EPA substantially increased the proportion of IL-1β^+^ IL-22^+^ keratinocytes, with further enhancement observed upon DGAT2i treatment (Figure 5E). Statistical analysis across experimental replicates confirmed the effects of EPA and DGAT2i on IL-1β and IL-22 expression (Figure 5F). However, IL-23 levels were barely detectable in keratinocytes by flow cytometry, suggesting its secretion into the supernatant rather than retention within the cells, which aligns with the ELISA results.

To assess whether the EPA+DGAT2i condition mimics a psoriatic keratinocyte phenotype, we compared it with untreated primary lesional epidermal keratinocytes (PLEKs) (Supplemental Figure 5C). We observed no significant difference in IL-1β expression, while IL-22 expression was significantly higher in untreated PLEKs. However, upon poly(I:C) stimulation, the difference in IL-22 expression between EPA+DGAT2i-treated cells and untreated PLEKs was no longer significant. These results indicate that EPA+DGAT2i alone is insufficient to fully mimic a psoriatic phenotype but can amplify inflammation driven by TLR3 activation.

Previous studies have shown that TLR3 serves as a pattern recognition receptor that detects double-stranded RNA and primarily participates in antiviral immune responses (39). Following activation, TLR3 recruits signaling molecules to initiate downstream pathways, including the NF-κB and MAPK signaling cascades (40). To investigate the involvement of these pathways, we performed WB analysis to examine MAPK and NF-κB activation in keratinocytes under various treatments. WB analysis showed that poly(I:C) stimulation led to increased phosphorylation of MAPK and NF-κB subunits. Sequential addition of EPA and DGAT2i further enhanced the phosphorylation levels of these proteins (Figure 5, G and H). Quantitative analysis (Supplemental Figure 5, D and E) revealed significant increases in p38 and p65 phosphorylation with poly(I:C) alone, while EPA primarily enhanced ERK, p38, and IκBα phosphorylation. DGAT2i further amplified phosphorylation of ERK, JNK, p38, and p65, underscoring its role in potentiating TLR3 pathway activation. These results demonstrate that EPA and DGAT2i amplify TLR3-mediated inflammatory responses by promoting phosphorylation of MAPK and NF-κB pathway components.

### Elevated DGAT2 secretion enhances CD8^+^ T cell activation, proliferation, and survival.

**Figure 6:**
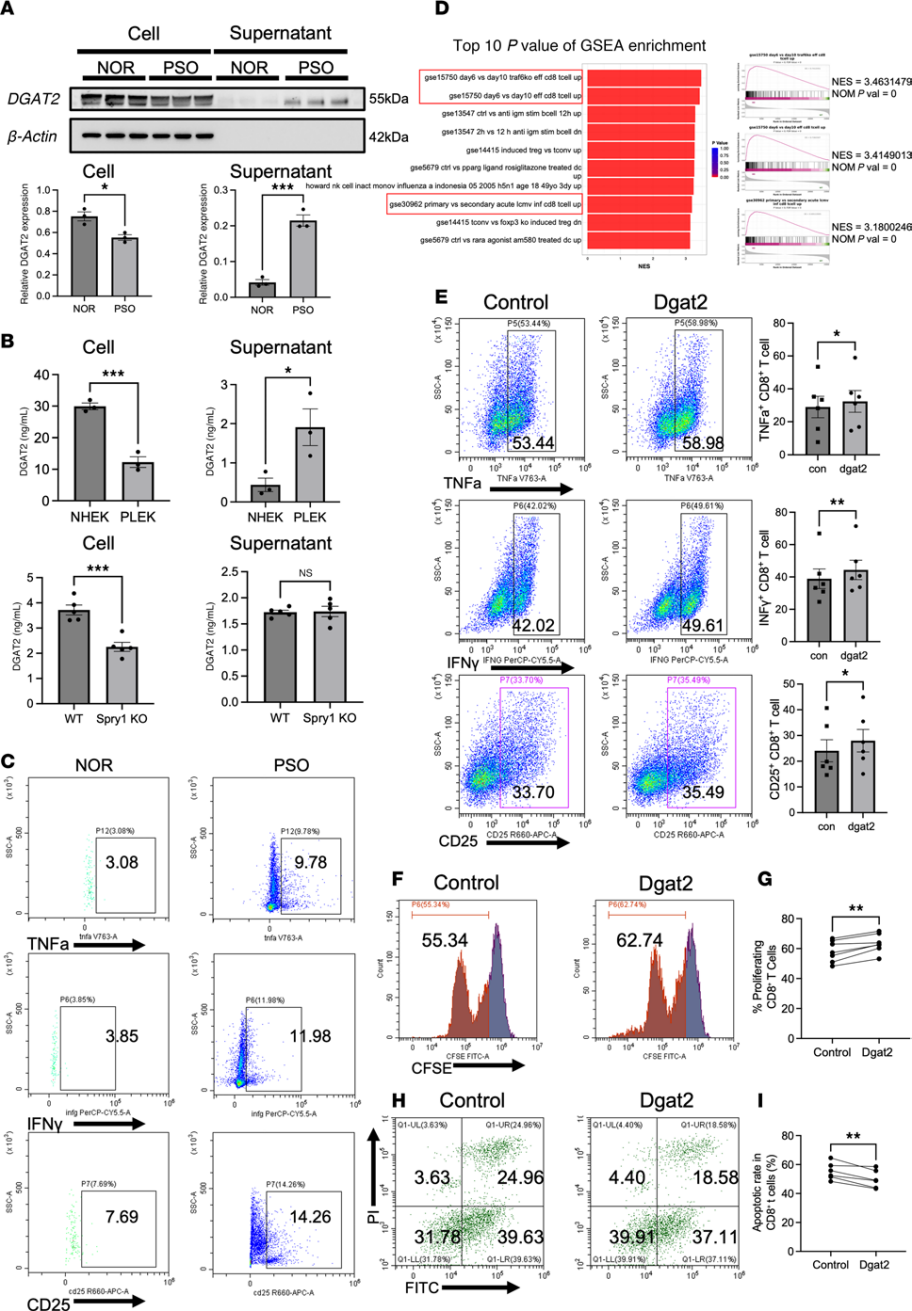
Elevated DGAT2 secretion enhances CD8^+^ T cell activation, proliferation, and survival. (**A**) Western blot analysis showing reduced intracellular DGAT2 expression in PSO compared with NOR, while secreted DGAT2 levels are paradoxically increased. *n* = 3 samples per group. (**B**) ELISA quantification of DGAT2 levels in cell lysates and supernatants from Spry1^ΔEpi^ and WT keratinocytes (*n* = 5 per group) and from psoriatic keratinocytes compared with normal controls (*n* = 3 per group). (**C**) Flow cytometry analysis of epidermal CD8^+^ T cells from PSO and NOR. (**D**) GSEA of RNA-sequencing data from Spry1^ΔEpi^ and WT mice epidermis that shows significant enrichment of immune-related pathways, particularly those associated with CD8^+^ T cells. NES, normalized enrichment score; NOM, nominal. (**E**) Flow cytometry analysis that reveals increased TNF-α, IFN-γ, and CD25 expression in CD8^+^ T cells treated with recombinant DGAT2 protein compared with controls. *n* = 6 samples per group. (**F** and **G**) CFSE proliferation assay that shows enhanced cell division in DGAT2-treated CD8^+^ T cells, as indicated by reduced fluorescence intensity. *n* = 7 samples per group. (**H** and **I**) Annexin V/PI staining indicates reduced apoptosis in DGAT2-treated CD8^+^ T cells. *n* = 6 samples per group. Paired 2-tailed Student’s *t* test was performed. *P < 0.05, **P < 0.01, ***P < 0.001.

We observed increased DGAT2 levels in the supernatant of keratinocytes derived from patients with psoriasis compared with healthy controls, despite reduced intracellular DGAT2 expression (Figure 6A). To further investigate this unexpected finding, we quantified DGAT2 levels in cell lysates and supernatants using ELISA (Figure 6B). The results verified that although DGAT2 secretion was elevated in psoriatic keratinocytes, the absolute concentration of DGAT2 in the supernatant remained much lower than its intracellular levels. Notably, in keratinocytes isolated from Spry1^ΔEPI^ mice, DGAT2 secretion did not significantly differ from wild-type controls, even though intracellular DGAT2 expression was reduced. These data suggest that DGAT2 secretion may be regulated independent of its intracellular abundance in psoriasis.

Flow cytometry analysis of epidermal samples from patients with psoriasis and healthy controls demonstrated that CD8^+^ T cells in lesional psoriatic skin exhibited markedly increased activation (elevated CD25 and CD69 expression), enhanced effector cytokine production (TNF-α and IFN-γ), and strengthened tissue-resident properties (CD103 and CXCR6) (Figure 6C and Supplemental Figure 6B). These findings are consistent with the well-established role of CD8^+^ T cells in driving psoriasis progression (41). RNA bulk sequencing data from the epidermis of Spry1^ΔEPI^ mice were analyzed using GSEA, focusing on the C7: Immunologic Signatures dataset from Molecular Signatures Database. The analysis revealed significant immune cell dysregulation, with a particular enrichment in pathways associated with CD8^+^ T cells (Figure 6D).

To investigate the effects of DGAT2 on CD8^+^ T cells, human peripheral blood-derived CD8^+^ T cells were stimulated with recombinant human DGAT2 protein. DGAT2 treatment significantly increased the expression of CD25, TNF-α, and IFN-γ (Figure 6E), indicating enhanced activation and effector function. Statistical analysis across independent experiments confirmed the significance of these effects. However, DGAT2 treatment did not significantly affect the expression of CD103, CXCR6, or CD69 (Supplemental Figure 6C). To assess the impact of DGAT2 on CD8^+^ T cell proliferation, a CFSE assay was performed. CD8^+^ T cells treated with DGAT2 exhibited a markedly higher proportion of cells with reduced fluorescence intensity, indicative of increased cell division (Figure 6F). Statistical analysis across replicates verified that DGAT2 significantly enhanced CD8^+^ T cell proliferation (Figure 6G). The effect of DGAT2 on CD8^+^ T cell survival was evaluated using apoptosis assays. DGAT2-treated CD8^+^ T cells showed a marked reduction in early apoptotic cells (annexin V–positive, PI-negative; Figure 6H) as well as late apoptotic and necrotic cells (annexin V–positive, PI-positive; Figure 6H). Statistical analysis demonstrated significant reductions in apoptosis following DGAT2 treatment (Figure 6I). These findings indicate that DGAT2 enhances the activation, proliferation, and survival of CD8^+^ T cells, which are critically involved in psoriasis pathogenesis.

To explore whether the effect of DGAT2 on CD8^+^ T cells could be explained by changes in lipid composition, we treated CD8^+^ T cells with EPA, diacylglycerol (DAG), or both (Supplemental Figure 6D). EPA treatment led to a significant increase in IFN-γ and CD25 expression, while TNF-α showed a modest but statistically nonsignificant increase. In contrast, DAG treatment did not alter CD8^+^ T cell activation markers. Neither EPA nor DAG affected CXCR6, CD103, or CD69 expression. These results suggest that EPA may partially contribute to CD8^+^ T cell activation, but the effects of DGAT2 cannot be fully recapitulated by lipid supplementation alone. This indicates that DGAT2 may influence CD8^+^ T cell function through additional mechanisms beyond direct lipid provision, such as modulating intracellular signaling.

## Discussion

In this study, we investigate the role of DGAT2 and lipid metabolism in the skin lesions of both a psoriasis-like mouse model (Spry1^ΔEpi^ mice) and patients with psoriasis. Our findings suggest a mechanism underlying psoriasis pathogenesis. Specifically, SPRY1 deficiency in keratinocytes downregulates DGAT2 expression in these cells and leads to lipid metabolism dysregulation. This triggers inflammatory events. In psoriatic keratinocytes, DGAT2 is not only reduced but also secreted at higher levels, which promotes CD8^+^ T cell activity and worse inflammation.

We found that in the lesional psoriatic skin, there are a substantial disruption of glyceride metabolism and a marked reduction in DGAT2 expression. A study has reported that Dgat2 gene was decreased in human psoriatic skin (42), while its role in psoriasis is largely unknown. DGAT2 deficiency in the epidermis causes skin abnormalities by impairing ceramide synthesis, essential for the skin barrier (16). Reduced DGAT2 expression could also contribute to atopic dermatitis (21), a chronic inflammatory skin disease. Beyond skin diseases, DGAT2 is also implicated in systemic inflammatory diseases, like metabolic dysfunction–associated steatohepatitis (MASH) (43), diabetes (44), and atherosclerosis (45). These findings highlight the potential importance of DGAT2 and lipid metabolism in skin immunity.

Using the Spry1^ΔEpi^ mouse model, we observed psoriasis-like features and significant lipid abnormalities. Lipid profiling showed disrupted glyceride, glycerophospholipid, and sphingolipid metabolism in the epidermis, with similar but less pronounced changes in plasma. We demonstrated that SPRY1 plays a critical role in regulating epidermal lipid metabolism. SPRY1 has been shown to influence key metabolic and inflammatory pathways. Knockdown of SPRY1 leads to significant downregulation of genes involved in glucose metabolism (*Cyp21a1*, *Cyp17a1*, *Col17a1*) and upregulation of inflammation-related genes, suggesting its dual role in metabolic and immune homeostasis (46). Loss of SPRY1 has been associated with metabolic disorders, such as metabolic syndrome, diabetes, and obesity, with a phenotype characterized by excessive adipogenesis and reduced bone mass (47). Additionally, SPRY1 modulates vascular and metabolic disease processes through its regulation of urokinase plasminogen activator receptor (48). The broader metabolic regulatory role of the Sprouty family is exemplified by SPRY4. It regulates critical pathways, such as ERK1/2 and Wnt/β-catenin signaling, which govern adipocyte differentiation, lipid metabolism, insulin sensitivity, and adipokine secretion (49). These findings highlight SPRY1’s role in psoriasis-related metabolic disturbances and inflammation.

We also found reduced DGAT2 expression in the epidermis of Spry1^ΔEpi^ mice and observed an interaction between SPRY1 and DGAT2. DGAT2 reduction may explain the lower TG and higher DAG in keratinocytes. As DGAT2 is a key enzyme in the final step of TG synthesis, it converts DAGs and FAs into TGs. We further investigate the role of DGAT2 and lipid metabolism abnormalities in the pathogenesis of psoriasis. Previous studies have shown that lipids markedly influence innate immunity by altering myeloid cell metabolism, especially in DCs. Lipids enhance TLR-mediated immune activation, increasing IL-23 production and driving a pro-inflammatory profile (34). TLR3 signaling has been shown to play a key role in psoriatic inflammation and keratinocyte-driven innate immunity (50). TLR3 activation also alters glucose and lipid metabolism and the production of inflammatory mediators. This highlights the close link between metabolic state and innate signaling (51, 52). Our findings align with these results, as EPA amplifies TLR3-mediated inflammatory responses in keratinocytes. Additionally, DGAT2 inhibitors exacerbate metabolic dysregulation and inflammation. TLR3 signals through TIR-domain-containing adapter-inducing interferon-β to activate 2 key pathways, NF-κB and IRF3/IRF7 (39), and also stimulates the MAPK cascade (40). We observed that EPA and DGAT2 inhibitors enhance TLR3-induced inflammation by activating NF-κB and MAPK pathways.

CD8^+^ T cells are central to psoriasis, producing pro-inflammatory cytokines such as IFN-γ and TNF-α (53). Our results highlight that keratinocytes from patients with psoriasis secrete increased levels of DGAT2. DGAT2 is classically described as an integral membrane protein of the endoplasmic reticulum. It is unexpected that DGAT2 would be detectable as a secreted protein. Previous studies have shown that membrane protein can be released from cells under stress or inflammatory conditions through microvesicles or exosomes (54, 55). Given that DGAT2 can relocalize from the ER to lipid droplets and mitochondria-associated membranes under metabolic stress (56, 57), we speculate that DGAT2 might be released via nonclassical pathways, such as exosomes, under certain conditions. This possibility remains open and needs further investigation. DGAT2 promotes CD8^+^ T cell proliferation and activation, increasing cytokine production while reducing apoptosis. This dual function in keratinocytes and immune cells underscores DGAT2’s importance in inflammation. Previous studies have shown that DGAT2 inhibition or overexpression can have both positive and negative effects depending on the context. In MASH, DGAT2 is an important therapeutic target, as its inhibition can reduce liver fibrosis but may also cause developmental toxicity during pregnancy in rodents (58). Emerging research also links DGAT2 to cancer. In obesity-related gastric cancer, DGAT2 promotes lipid droplet formation in adipocytes and helps cancer cells resist anoikis, facilitating metastasis (59). Similarly, inhibiting DGAT2 in cancer cells, like MCF-7 breast cancer cells, enhances their sensitivity to radiation (60). Conversely, DGAT2 may function as a tumor suppressor in certain cancer types, such as HCC and melanoma (18, 61). Overexpressing DGAT2 in HCC cells reduces proliferation and alters cell cycle genes, and its low expression correlates with poor prognosis. These findings highlight DGAT2’s diverse roles in disease, emphasizing the need to explore its therapeutic potential further.

A limitation to consider is that DGAT2 mRNA is expressed in the sebaceous glands of normal human skin, where it may help produce TGs and sebum (42). Prior studies demonstrate that changes in DGAT2 levels can affect sebocyte lipid metabolism and skin barrier function (62, 63), which means DGAT2 may have roles beyond keratinocytes. In our study, we did not specifically analyze DGAT2 in sebaceous glands, because our model used K14-Cre mice to delete *Spry1* only in keratinocytes. K14-Cre is not active in sebocytes, so the effects we observed are mainly due to changes inside keratinocytes. While lipid metabolism in sebaceous glands may influence skin inflammation and barrier function, this aspect was not addressed in our current work. Future studies using sebocyte-specific models or direct lipid profiling of sebaceous glands will be essential to define DGAT2’s role in these cells.

In conclusion, we found that Spry1^ΔEpi^ mice developed psoriasis-like symptoms and used this model to investigate the crosstalk between lipid metabolism and immune pathways in the pathogenesis of psoriasis. We observed decreased DGAT2 expression and abnormal glyceride metabolism in the psoriatic lesions of Spry1^ΔEpi^ mice and patients with psoriasis. Notably, Spry1^ΔEpi^ mice also exhibited systemic lipid metabolism dysregulation, including glycerides, glycerophospholipid, sphingolipid, and FA. The disruption of DGAT2 and lipid metabolism led to immune dysfunction in keratinocytes and CD8^+^ T cells, further promoting inflammatory responses. Our study highlights the critical role of DGAT2 and lipid metabolism in the pathogenesis of psoriasis.

## Methods

### Sex as a biological variable.

Sex as a biological variable was not initially considered in this study. Both male and female mice, as well as patients, were included in the analysis.

### Mice.

Epidermal keratinocyte-specific Spry1-knockout (Spry1^ΔEpi^) mice were previously described (26). The mice were bred at the Laboratory Animal Center of the Second Affiliated Hospital of Zhejiang University. To induce Cre recombination, tamoxifen (MedChemExpress) was administered orally at a dose of 0.1 mg/g body weight, using a 20 mg/ml solution in corn oil. The treatment was given 4 times per week for 5 weeks. Whenever possible, mice were randomly assigned to experimental groups, except for experiments requiring specific genotypes, where sex-matched littermate controls were used.

### Epidermal keratinocyte culture.

Primary human epidermal keratinocytes, including normal human epidermal keratinocytes (NHEKs) and PLEKs, were isolated from skin samples obtained at the Second Affiliated Hospital, Zhejiang University School of Medicine, as previously described. Primary epidermal keratinocytes from both human and mouse skin were isolated using the same procedure (25). Skin samples were digested overnight at 4 °C using Dispase medium (Gibco, #17105041). To prepare a single-cell suspension, the separated epidermis was further digested with 0.25% trypsin (Thermo Fisher Scientific, #15050065). The digested cells were neutralized with fetal bovine serum, centrifuged at 1000 rpm for 5 minutes, and then cultured in KC culture medium (Lonza, #CC-3107) in a humidified incubator set at 37 °C with 5% carbon dioxide. The culture medium was refreshed every two days. To induce SPRY1 knockout in KCs in vitro, 4-hydroxy tamoxifen (2.5 nM) was added to the KC culture medium for three consecutive days.

### Isolation of peripheral blood mononuclear cells (PBMCs).

To prepare PBMCs, fresh anticoagulated blood (2 mL) was collected from donors and diluted 1:1 with calcium- and magnesium-free PBS or another suitable buffer. Next, 3 mL of Lymphocyte Separation Medium (Biosharp, #BL590) was added to a centrifuge tube, and the diluted blood was carefully layered on top of the medium. The tube was centrifuged at 750 × *g* for 25 minutes at room temperature, with acceleration and deceleration both set to 3. After centrifugation, 4 layers of cells were visible from top to bottom: the plasma layer, a ring-shaped milky layer of lymphocytes, the transparent separation medium, and the red blood cell layer. The lymphocyte layer (second layer) was carefully transferred to a new tube, mixed with 3 times the volume of PBS, and centrifuged at 250 × *g* for 10 minutes. The supernatant was discarded, and the cell pellet was resuspended in 1 mL of PBS (2% fetal bovine serum).

### Human peripheral CD8^+^ T cell isolation and stimulation.

Human purified CD8^+^ T cells were isolated using the MojoSort™ Whole Blood Human CD8 T Cell Isolation Kit (BioLegend, #480164). To start, 1 mL of the previously resuspended PBMC solution was aliquoted into a 5 mL (12 × 75 mm) polypropylene tube. Then, 20 μL of the Biotin-Antibody Cocktail was added, mixed thoroughly, and incubated at room temperature for 5 minutes. The streptavidin nanobeads were resuspended by vortexing at maximum speed (5 quick taps). Next, 20 μL of streptavidin nanobeads was added to the tube, mixed well, and incubated at room temperature for another 5 minutes. Afterward, 2.5 mL of MojoSort™ Buffer was added for the first separation. The tube was placed in a magnetic separator for 5 minutes, and the unlabeled fraction was collected. The labeled cells were resuspended in 3.5 mL of MojoSort™ Buffer for a second separation. The tube was again placed in the magnetic separator for 5 minutes, and the unlabeled fraction was collected.

The collected cell suspension was centrifuged at 300 × *g* for 10 minutes at room temperature. The cell pellet was resuspended and activated at 37 °C with 5% CO_2_ for 72 hours using T Cell TransAct™ (Miltenyi Biotec, #130-111-160) at a 1:100 dilution in ImmunoCult™-XF T Cell Expansion Medium (Stemcell, #10981) supplemented with Human IL-2 (20 IU/mL). After the initial activation, any residual reagents were removed by centrifuging the cells at 300 × *g* for 10 minutes and aspirating the supernatant. Fresh supplemented TexMACS Medium (Miltenyi Biotec, #130-097-196) (1 mL) was then added, and the cells were incubated at 37 °C with 5% CO2. Every 2 days, the cell suspension was split into 2 equal parts, and fresh supplemented TexMACS Medium was added. On day 14, the cells were used for downstream applications.

For subsequent assays, CD8^+^ T cells were seeded in 12-well plates at a density of 1 × 10^5^ cells per well. Recombinant DGAT2 protein (Meilian, #ml092027) was added to each well at a concentration of 200 ng/mL to stimulate the CD8^+^ T cells for 24 hours. These cells were then used for proliferation, apoptosis, and functional assays.

### Cell proliferation assay.

Primary human CD8^+^ T cells were labeled with 5 μM CFSE (Invitrogen, #C34554) following the manufacturer’s instructions. The labeled cells were then plated in a 12-well plate at a density of 1 × 10^5^ cells and stimulated with recombinant DGAT2 protein (Meilian, #ml092027) at a concentration of 200 ng/mL. After 24 hours of stimulation, the cells were collected and stained with Live/Dead Zombie UV Dye (BioLegend, #423108). Data acquisition was performed using a Beckman CytoFlex LX flow cytometer.

### Apoptosis assay.

For apoptosis analysis, cells were stained using an Annexin V-FITC Apoptosis Kit (Beyotime Biotechnology, #C1062S) following the manufacturer’s instructions. Primary human CD8^+^ T cells were seeded in a 12-well plate at a density of 1 × 10^5^ cells and stimulated with DGAT2 at a concentration of 200 ng/mL for 24 hours. After stimulation, the cells were centrifuged at 300 × *g* for 10 minutes, the supernatant was discarded, and the cell pellet was gently resuspended in PBS. The cells were centrifuged again at 300 × *g* for 10 minutes, the supernatant was removed, and 195 μL of Annexin V-FITC binding buffer was added to gently resuspend the cells. Next, 5 μL of Annexin V-FITC and 10 μL of propidium iodide (PI) staining solution were added, mixed gently, and incubated in the dark at room temperature (20–25°C) for 10–20 minutes. The cells were then placed on ice. Data acquisition was performed using a Beckman CytoFlex LX flow cytometer.

### RNA isolation and quantitative real-time PCR.

Total RNA was extracted from the samples using FastPure Cell/Tissue Total RNA Isolation Kit V2 (Vazyme, #RC112-01). One microgram of total RNA was used to synthesize cDNA with HiScript III All-in-one RT SuperMix Perfect for qPCR (Vazyme, #R333-01). Real-time RT-PCR was performed using FastStart Universal SYBR Green Master Mix (ROX) (Roche Life Science, #04913914001) following the manufacturer’s instructions. Primer sequences are listed in Supplemental Table 5. Gene expression was calculated using the 2–ΔΔCT method with β-actin as the reference gene.

### Western blot analysis.

Protein levels were measured using a BCA protein assay kit (Thermo Fisher Scientific, 23225). Proteins were separated by SDS-PAGE and transferred onto a nitrocellulose membrane (MilliporeSigma). The membrane was blocked with 5% skim milk in TBS-T (20 mM Tris-HCl, pH 7.5, 137 mM NaCl, and 0.05% Tween 20) for 1 hour at room temperature, followed by overnight incubation with primary antibodies (details provided in Supplemental Table 4). The next day, the membrane was incubated with HRP-conjugated secondary antibodies (1:5000 dilution) (Supplemental Table 3) and visualized using ECL Substrate (Thermo Fisher Scientific, #46641).

### Histology and immunofluorescence.

Paraffin-embedded skin sections were obtained from healthy individuals and patients with psoriasis. For histology, 8-μm-thick skin sections were stained with H&E. For immunofluorescence staining, tissues were embedded in optimal cutting temperature compound. Six-micrometer-thick skin sections were fixed in 4% paraformaldehyde, permeabilized for 10 minutes with 0.1% Triton X-100, and blocked with 3% BSA (Sigma-Aldrich, A9418). The sections were then incubated with primary antibodies, followed by Alexa 488/555–conjugated secondary antibodies (1:1000) for 1 hour at room temperature, with 3 PBS washes in between. Finally, the sections were stained with DAPI, mounted with antifade mounting medium (Solarbio), and imaged using a fluorescence microscope (Leica DM5500B).

### Lipid analysis by Nile red.

A stock solution of Nile red (1 mg/mL; Sigma-Aldrich) in acetone was diluted to a 1 μg/mL working solution in a 75:25 mixture of glycerol and water, then mixed thoroughly by vortexing. Frozen skin sections from mice and humans were thawed at room temperature for 5 minutes, briefly rinsed with PBS, and stained with the working solution in the dark for 30 minutes. Afterward, the sections were stained with DAPI for 10 minutes, and coverslips were applied.

For staining primary human and mouse keratinocytes, cells were cultured on coverslips, washed once with PBS, and stained following the same protocol as used for skin sections. Images were taken using a fluorescence microscope (Leica DM5500B).

### ELISA.

Cell supernatants were collected from primary keratinocytes of healthy individuals, patients with psoriasis, Spry1-knockout mice, and wild-type mice. When the keratinocytes reached 60% confluence, stimulants were added to the culture medium, and the supernatant was collected after 48 hours. The concentrations of diacylglycerol, triglyceride, and IL-23 in the supernatant were measured using ELISA kits (Cloud-Clone Corp, #CEC038Ge; Nanjing Jiancheng Bioengineering Institute, #A110-1-1; UpingBio, #SYP-M0194) according to the manufacturer’s instructions.

### Plasmid construction and transfection.

The pCMV-Dgat2(mouse)-3×HA-Neo plasmid encodes the mouse DGAT2 gene with 3 HA tags for detection. Similarly, the pCMV-Spry1(mouse)-3×FLAG-Neo plasmid was designed to express the mouse Spry1 gene fused with a 3×FLAG tag. Plasmids were obtained from MiaoLingBio, China. Both plasmids feature a CMV promoter to ensure strong gene expression in mammalian cells and a neomycin resistance (NeoR/KanR) cassette for cell selection. The backbone includes an SV40 origin for replication in mammalian cells, an AmpR gene for bacterial selection, and multiple cloning sites with restriction enzyme recognition sites, such as KpnI, HindIII, and EcoRI, for easy gene manipulation. Additionally, these plasmids incorporate a WPRE element to enhance expression levels and an HSV TK poly(A) signal for proper transcription termination.

We seeded cells in a 6-well plate 1 day before transfection to ensure they reached 70%–90% confluence. To prepare the DNA-transfection reagent complexes, we diluted 5 μg of plasmid DNA per well in Opti-MEM™ medium (Thermo Fisher Scientific, #31985070). Separately, we diluted Lipofectamine™ 3000 (Thermo Fisher Scientific, #L3000015) in Opti-MEM™ medium according to the manufacturer’s instructions. We combined the diluted DNA and transfection reagent in a 1:1 ratio, mixed gently, and incubated at room temperature for 20 minutes to allow complex formation. We removed the existing growth medium from the cells and replaced it with Opti-MEM™ medium. We carefully added the DNA-transfection reagent complexes dropwise to the cells, ensuring even distribution, and gently rocked the plate to mix. We incubated the cells with the complexes at 37°C in a humidified incubator with 5% CO_2_ for 24 hours. After incubation, we replaced the medium with KC culture medium (Lonza, #CC-3107) and continued to incubate the cells at 37°C for an additional 48 hours before analyzing them for gene expression or other experiments.

### Flow cytometric analysis.

The skin sample was digested overnight at 4 °C using Dispase medium (Gibco, #17105041). For the preparation of a single-cell suspension, the epidermis was separated and further digested with 0.25% trypsin (Thermo Fisher Scientific, #15050065). After digestion, the epidermal cells were neutralized with fetal bovine serum and centrifuged at 450 *g* for 5 minutes. The cells were then stimulated with resiquimod (MedChemExpress, #HY-13740) and a Cell Activation Cocktail (with Brefeldin A) (BioLegend, #423304) for 4.5 hours. Following stimulation, the suspension was centrifuged at 450 *g* for 5 minutes and resuspended in 100 μl of vehicle (PBS/2% fetal bovine serum) containing FcR block (BioLegend, #S17011E). Cells were then incubated with Live/Dead Zombie dye (BioLegend, #423107) for 15 minutes in the dark. For functional analysis of CD8^+^ T cells, the single-cell suspension from the epidermis was incubated with antibodies specific to CD45, CD8, CD25, CD69, CD103, and CXCR6 for 30 minutes in the dark. After incubation, the cells were fixed and permeabilized (Thermo Fisher Scientific, #00552300), followed by staining for TNF-α and IFN-γ. To assess keratinocyte function, the keratinocytes were fixed and permeabilized (Thermo Fisher Scientific, #00552300) and stained for IL-1β, IL-6, IL-17, IL-22, IL-23, and TNF-α. All antibodies were diluted at a 1:100 ratio for staining. The samples were analyzed using a Beckman CytoFlex LX flow cytometer with CytExpert software. Detailed antibody information can be found in Supplemental Table 1.

### Immunoprecipitation.

The mouse keratinocytes were transfected with plasmids encoding FLAG-spry1, HA-dgat2, or both, as indicated. After 72 hours, cells were washed with PBS and lysed in RIPA Complete Lysis Buffer (Beyotime Biotechnology, #P0038-100ml) supplemented with Protease and Phosphatase Inhibitors (Thermo Fisher Scientific, #78442) to extract the proteins. The lysates were then incubated with anti-FLAG antibody-conjugated beads (AlpalifeBio, #KTSM1338) at 4°C for 4 hours with gentle rotation to allow immunoprecipitation of FLAG-tagged spry1. Following extensive washing with lysis buffer to remove unbound proteins, the beads were subjected to magnetic separation, resuspended in 1× loading buffer, and boiled for 5 minutes before western blot analysis.

### Non-targeted lipid metabolism profiling.

Metabolites were extracted from the samples using organic reagents for protein precipitation, simultaneously preparing quality control (QC) samples by mixing equal volumes of the experimental samples. The extracted samples were then analyzed using a random sample order, with QC samples inserted at the beginning, middle, and end of the sample sequence for technical replicate evaluation. The samples underwent both positive and negative ion scanning in mass spectrometry. The raw mass spectrometry data were converted into readable mzXML format using the MSConvert software from Proteowizard. Peak extraction was performed using XCMS software, followed by quality control of the extracted peaks. The extracted compounds were annotated for adduct ions using CAMERA software, and primary identification was performed using metaX software. Identification was conducted with both primary mass spectrometry data and secondary mass spectrometry data, which were matched against an in-house standard database. Candidate metabolites were annotated using databases such as HMDB, KEGG, and others, providing information on the metabolites’ physicochemical properties and biological functions. MetaX software was used to quantify the differential metabolites and screen for significant metabolic differences.

### Chromatin immunoprecipitation (ChIP).

Chromatin immunoprecipitation was performed using epidermal tissue from mouse skin. The tissue was immediately frozen in liquid nitrogen and ground to a fine powder before further processing. ChIP assays were carried out using the Chromatin Immunoprecipitation (ChIP) Assay Kit (Millipore, #17-295), following the manufacturer’s instructions with minor adjustments for epidermal tissue. Briefly, chromatin was cross-linked by incubating the tissue homogenate with 1% formaldehyde at 37°C for 10 minutes. The reaction was quenched, and the material was washed with ice-cold PBS containing protease inhibitors. The pellet was resuspended in SDS lysis buffer and incubated on ice for 10 minutes. Chromatin was sheared to an average size of 200–1000 bp using a Cole Parmer ultrasonic processor (50 W, 2 mm probe, 30% amplitude) with 3–4 cycles of 10-second pulses, while maintaining the sample on ice.

After centrifugation at 12,000 *g*, the supernatant was diluted 10-fold with ChIP dilution buffer and pre-cleared with protein A agarose/salmon sperm DNA slurry. Immunoprecipitations were performed overnight at 4°C using specific antibodies or no-antibody controls. Complexes were captured with protein A agarose beads, washed sequentially with low-salt, high-salt, LiCl, and TE buffers, and eluted in SDS/NaHCO_3_ elution buffer. Cross-links were reversed at 65°C for 4 hours, followed by proteinase K digestion. DNA was purified by phenol-chloroform extraction and ethanol precipitation, then resuspended for downstream PCR analysis. DNA primers used are listed in Supplemental Table 2.

### Cycloheximide chase assay.

To assess protein stability, a cycloheximide chase assay was performed. Primary mouse keratinocytes were cultured under standard conditions and treated with cycloheximide (MCE, #HY-12320) at a final concentration of 20 uM to inhibit de novo protein synthesis. Cells were harvested at multiple time points (0, 2, 4, and 8 hours) following CHX treatment for protein extraction. After lysis in RIPA buffer containing protease inhibitors, total protein was collected and analyzed by western blotting to assess protein levels.

### Statistics.

Statistical analyses were conducted using GraphPad Prism 10.2.3. Unpaired 2-tailed Student’s t tests were used to compare differences between 2 groups, and 1-way analysis of variance (ANOVA) followed by Tukey’s post hoc test was used to compare differences between multiple groups. Data are presented as the mean ± SD, unless stated otherwise. A *P*-value of < 0.05 was considered statistically significant (*P* > 0.05 was considered nonsignificant).

### Study approval.

The collection of patient samples was approved by the Ethics Committee of the Second Affiliated Hospital of Zhejiang University School of Medicine (approval number 2022-1062). All participants provided written informed consent for the procedures involved. All animal experiments were approved by the Animal Ethics Committee of the Second Affiliated Hospital of Zhejiang University School of Medicine (2020.NO.101) and conducted following the committee’s guidelines.

### Data availability.

All data associated with this study are present in this article or in the supplement, and values for all data points in graphs are reported in the Supporting Data Values file. The raw bulk RNA-Seq and scRNA-Seq data of mice generated in our study are deposited in the Gene Expression Omnibus (GEO) database under accession numbers GSE289141 and GSE289142. The datasets used and/or analyzed in the current study are available from the corresponding author upon reasonable request.

## Author contributions

XYM and YYL performed conceptualization; YZC and FX performed data curation; YZC and FX performed formal analysis; XYM acquired funding; YYL, LRY, YZC, FX, XBC, FFZ, YL, and YXZ investigated; YYL and LRY developed methodology; XYM and YYL performed project administration; XYM supervised; YYL, LRY, YZC, and FX prepared the original draft; and YYL and XYM performed review and editing.

## Supplementary Material

Supplemental data

Unedited blot and gel images

Supporting data values

## Version 1 07/22/2025

In-Press Preview

## Version 2 09/09/2025

Electronic publication
