# Supplementary material for: DGAT2 reduction and lipid dysregulation drive psoriasis development in keratinocyte-specific SPRY1-deficient mice
Source: JCI Insight. 2025 Jul 22;10(17):e192507. doi: 10.1172/jci.insight.192507 (PMC12487672; doi:10.1172/jci.insight.192507)
Supplement: Supplemental data [file jciinsight-10-192507-s058.pdf]

SUPPLEMENTARY FIGURE

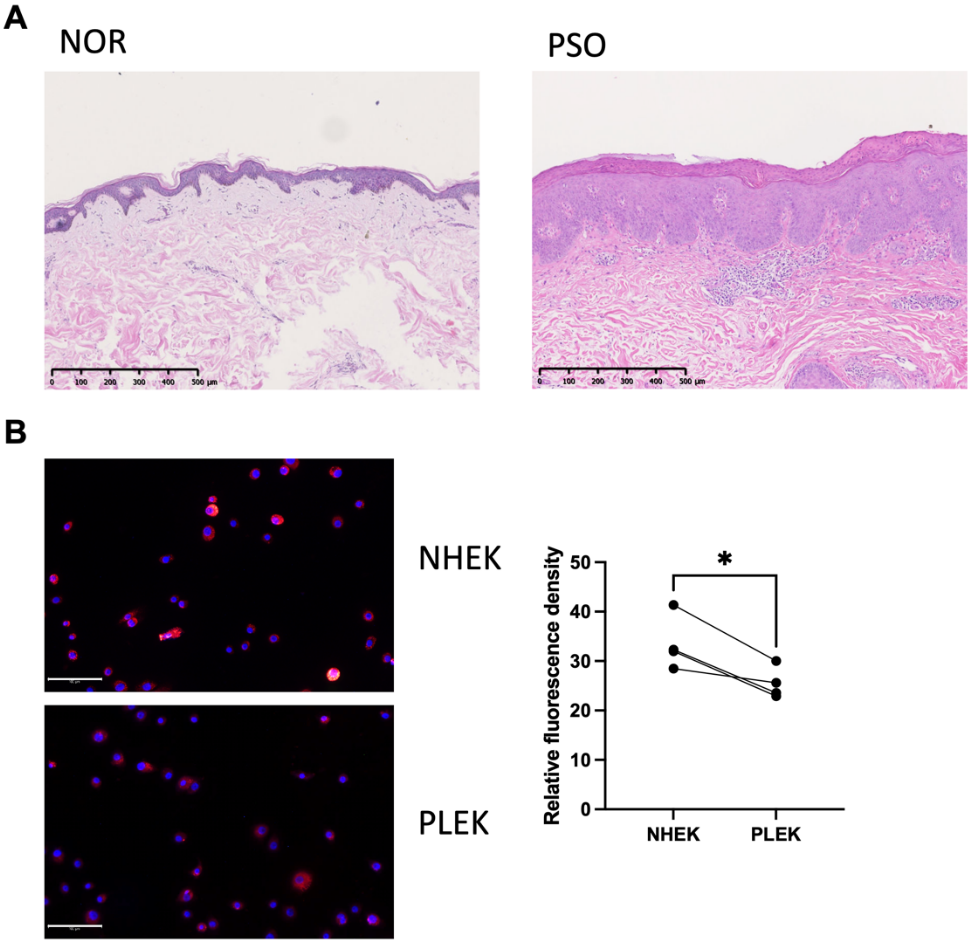

Supplementary Figure S1 Histological and TG Staining Analysis in Healthy and Lesional Psoriatic Skin. (A) Histological analysis using H&E staining of healthy (NOR) and psoriatic (PSO) skin samples. Lesional psoriatic skin shows marked epidermal hyperplasia and inflammation. Scale bars in histological images = 500 µm. (B) TG staining in keratinocytes isolated from healthy individuals (NHEK) and psoriasis patients (PLEK). Scale bars = 50 µm. n = 4 samples per group.

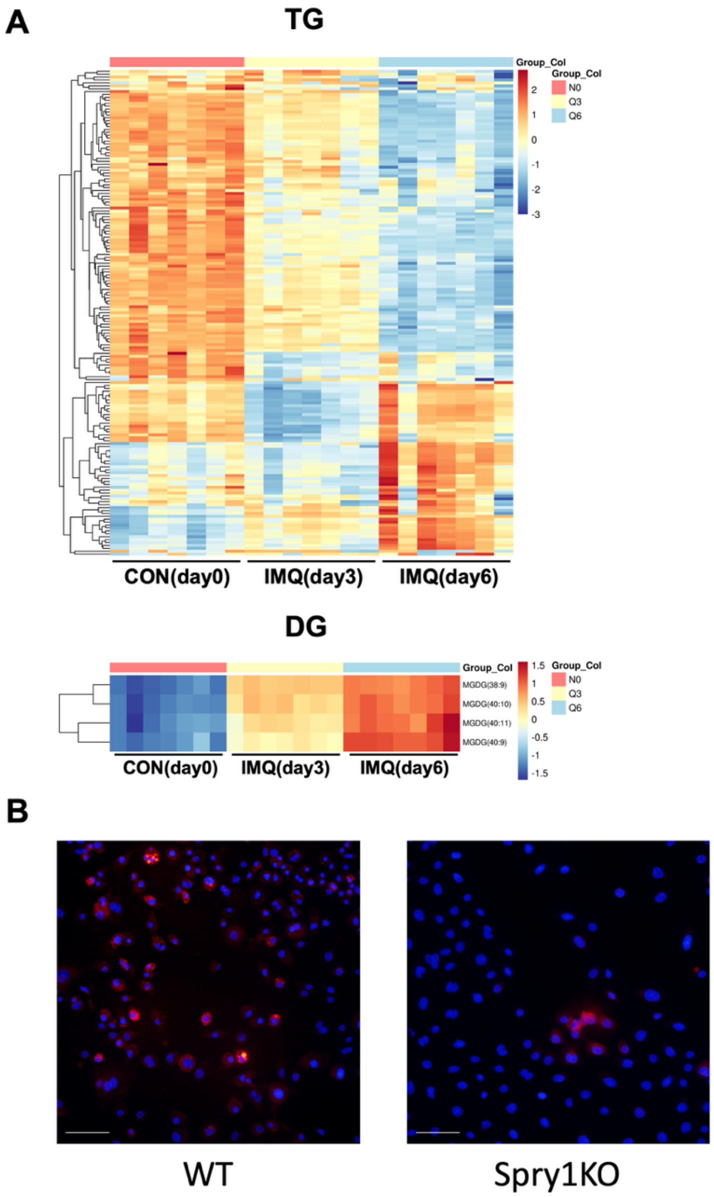

Supplementary Figure S2 Altered Glyceride Levels in Psoriasis-like Skin and SPRY1-deficient Keratinocytes. (A) Heatmaps showing TG and DG in wild-type mice and the IMQ-induced psoriasis-like mouse model across different IMQ induction times (CON(day0), IMQ(day3), and IMQ(day6)). (B) TG staining in keratinocytes isolated from wild-type (WT) controls and Spry1$^{\Delta Epi}$ (Spry1KO) mice.

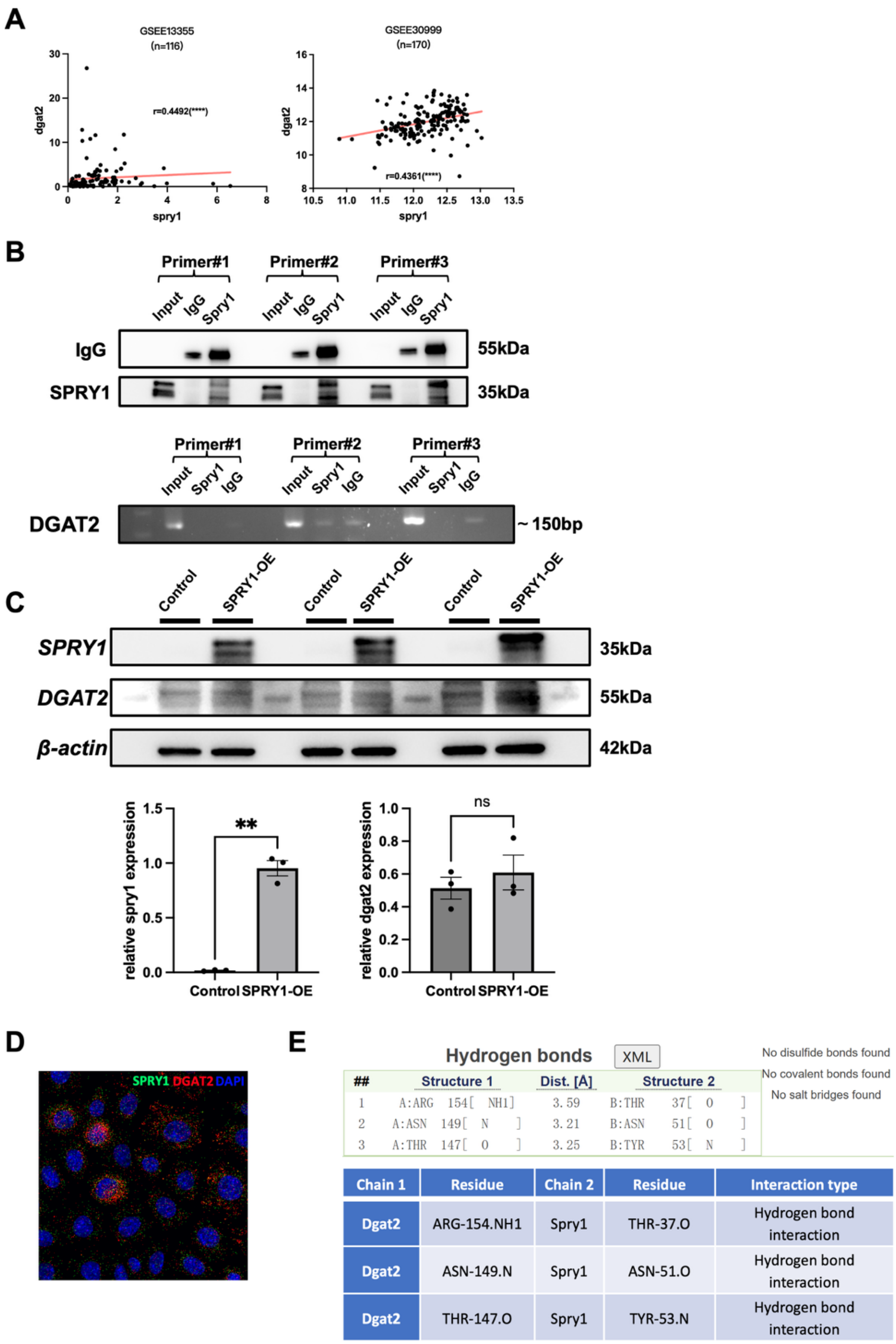

Supplementary Figure S3 SPRY1 is associated with DGAT2 expression. (A) Positive correlation between SPRY1 and DGAT2 expression in psoriatic skin (GSE13355, GSE30999). (B) Chromatin immunoprecipitation (ChIP) assay in mouse

keratinocytes showing that SPRY1 does not bind to the DGAT2 promoter region. (C) Western blot analysis showing that overexpression of SPRY1 in primary keratinocytes did not increase DGAT2 protein levels. n = 3 samples per group. (D) Immunofluorescence staining shows colocalization of SPRY1 and DGAT2 in keratinocytes. (E) Molecular modeling predicts hydrogen bonds between specific residues of SPRY1 and DGAT2, forming a stable interaction interface. Two-tailed Student's t test was performed. **$P < 0.01$, ****$P < 0.0001$.

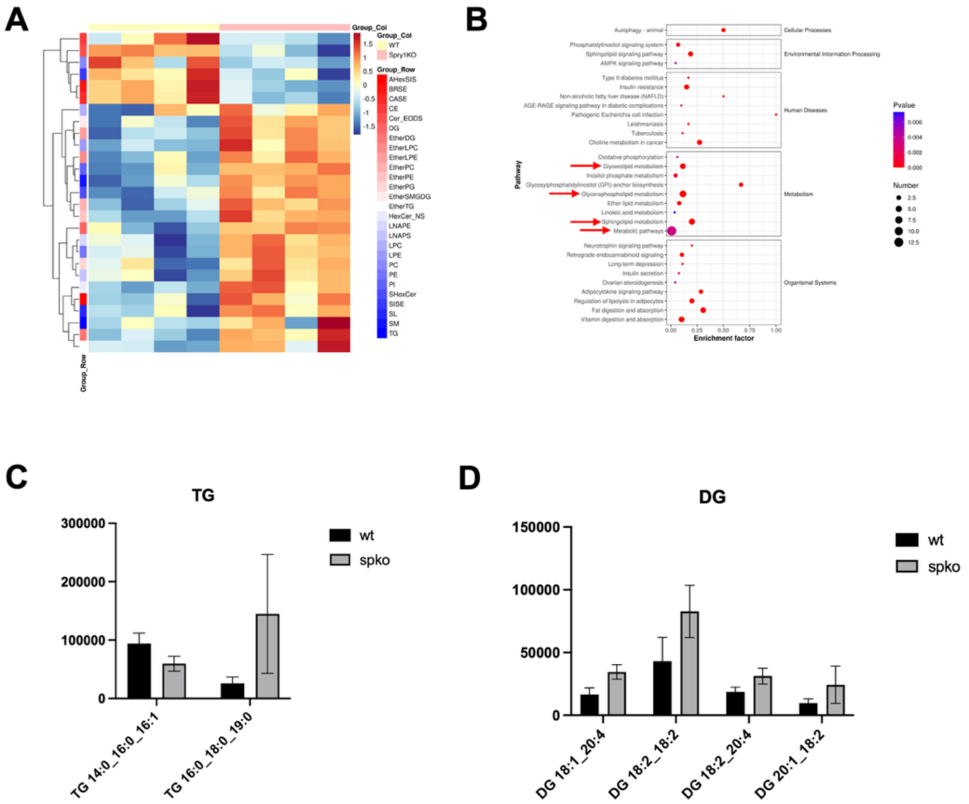

Supplementary Figure S4 Systemic lipid alterations in plasma of Spry1$^{\Delta Epi}$ mice. (A) Heatmap analysis of plasma lipids showing changes in lipid classes, including phospholipids and glycerides, in Spry1$^{\Delta Epi}$ mice compared to WT. (B) Pathway enrichment analysis showing disruptions in lipid metabolism, consistent with epidermal findings. (C–D) Quantitative analysis showing moderate changes in triglycerides and diacylglycerols in plasma, with less pronounced effects compared to epidermis. For lipid profiling, n = 4 mice per genotype.

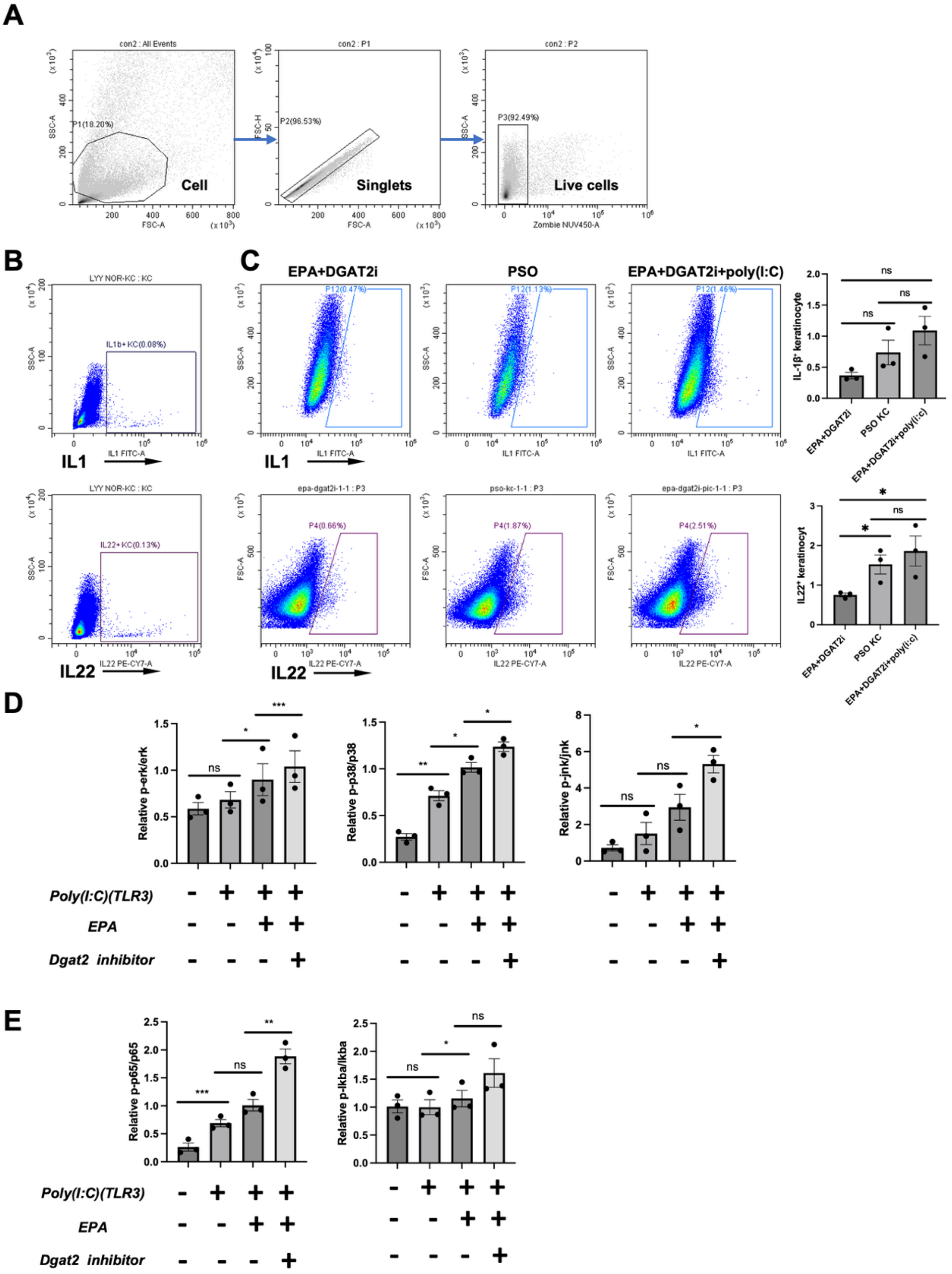

Supplementary Figure S5 Keratinocyte cytokine production and MAPK/NF-κB pathway activation are enhanced by lipid dysregulation under TLR3 stimulation. (A) Gating strategy for identifying cultured single live keratinocytes. (B) IL-1β⁺ and IL-22⁺ keratinocytes can be detected in human epidermis. (C) Flow cytometry analysis

showing differential expression of IL1B and IL22 between EPA+DGAT2i-treated keratinocytes and untreated primary lesional epidermal keratinocytes (PLEKs); upon poly(I:C) stimulation, the difference in IL22 expression was no longer significant. n = 3 samples per group. (D, E) Quantitative analysis of MAPK and NF-κB pathway activation from Western blot experiments confirming significant increases in phosphorylation of ERK, JNK, p38, p65, and IκBα with poly(I:C), EPA and DGAT2i treatment. For Western blotting, 20 μg of protein is loaded per well. n = 3 samples per group. Two-tailed Student's t test was performed. $^{*}P < 0.05$, $^{**}P < 0.01$, $^{***}P < 0.001$.

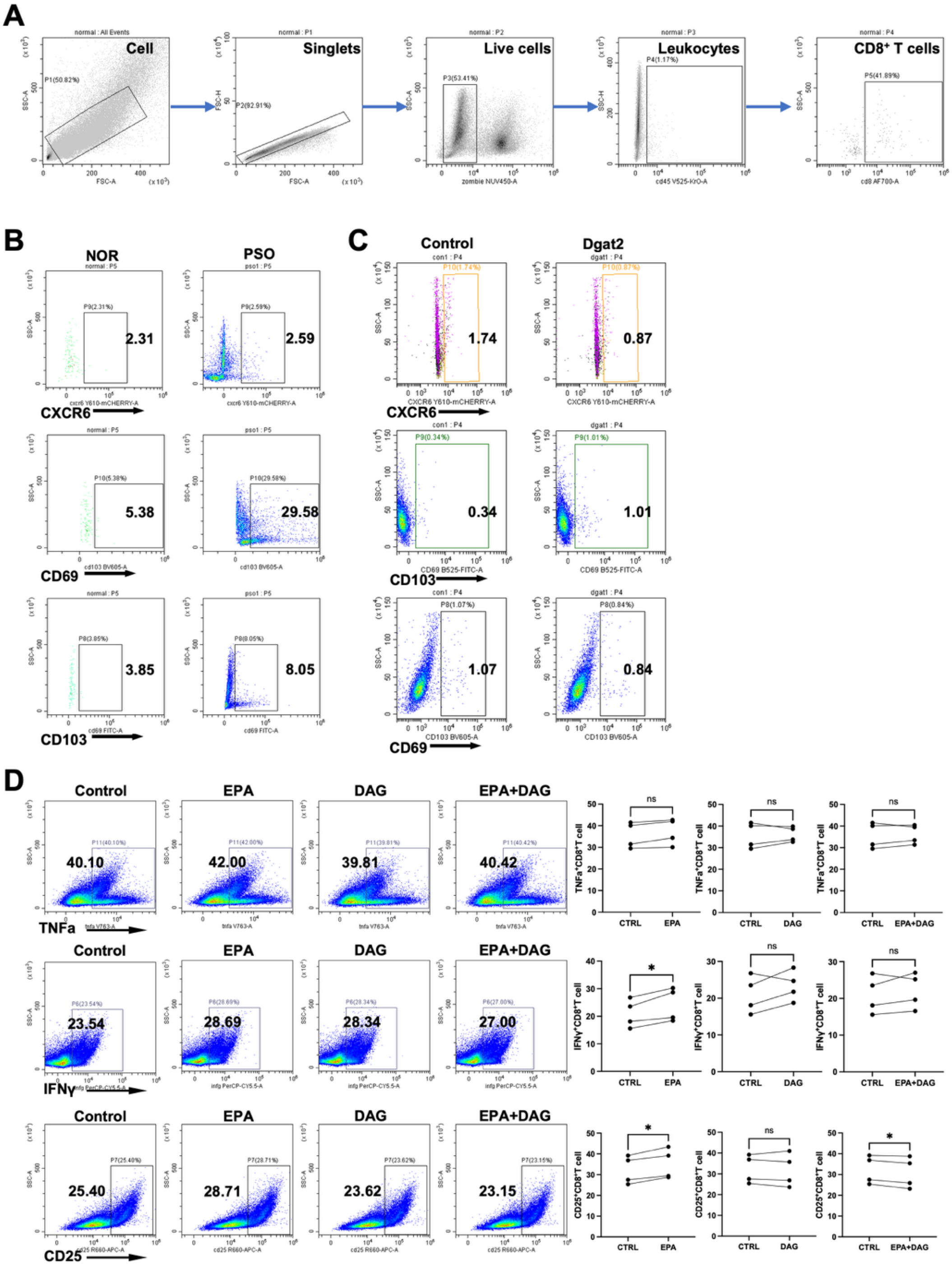

Supplementary Figure S6 Flow cytometry reveals increased tissue-resident markers in psoriatic epidermal CD8⁺ T cells and minimal effects of lipid treatments on marker expression in vitro. (A) Gating strategy for identifying cultured single live CD8⁺ T cells. (B) Flow cytometry analysis of epidermal CD8⁺ T cells from psoriasis patients and healthy controls showing increased expression of

CD103, CXCR6, and CD69 in psoriatic samples. (C) DGAT2-treated $CD8^+$ T cells showing no significant changes in tissue-resident markers CD103, CXCR6, or CD69. (D) Flow cytometry analysis of $CD8^+$ T cells following treatment with EPA, DAG, or a combination of both. n = 4 samples per group. Two-tailed Student's t test was performed. *$P < 0.05$.

SUPPLEMENTARY TABLE

Flow Cytometry Antibody Table (Table 1)

Reagent name	Company	Catalog number
IL-1 beta (Pro-form) Monoclonal Antibody	Invitrogen	#12-7114-82
IL-17A Monoclonal Antibody	Invitrogen	#25-7177-82
PerCP/Cyanine5.5 anti-mouse IL-22 Antibody	BioLegend	#516411
APC anti-mouse IL-12/IL-23 p40	BioLegend	#505206
Brilliant Violet 650™ anti-mouse TNF-α Antibody	BioLegend	#506333
Brilliant Violet 510™ anti-human CD45 Antibody	BioLegend	#368526
Alexa Fluor® 700 anti-human CD8 Antibody	BioLegend	#344724
APC anti-human CD25	BioLegend	#985810
Alexa Fluor® 488 anti-human CD69 Antibody	BioLegend	#310916

PE/Dazzle™ 594 anti-human CD186 (CXCR6) Antibody	BioLegend	#356016
Brilliant Violet 605™ anti-human CD103 (Integrin αE) Antibody	BioLegend	#350218
PerCP/Cyanine5.5 anti-human IFN-γ Antibody	BioLegend	#502526
Brilliant Violet 785™ anti-human TNF-α Antibody	BioLegend	#502948
PE/Cyanine7 anti-human 1L-22 Antibody	BioLegend	#366707
FITC anti-human IL-1 beta Antibody	BioLegend	#511705
PE anti-human IL-17A Antibody	BioLegend	#512306
IL-23 p19 Monoclonal Antibody	Invitrogen	#50-7823-42

66 **DNA Primers Table (Table 2)**

Gene Name	Primer Type	Sequence (5' → 3')
Dgat2	Forward Primer1	CCACTCATCATGCAAGTGTTTC
	Reverse Primer1	CACCTTTGGGTTGCATACTGT

Forward Primer2	AGCCTAGTCTACATAGTGCCAG
Reverse Primer2	CAGGGTGTCTTTTGTTTGGCT
Forward Primer3	GTCAGGTCCAGCTGTAACCT
Reverse Primer3	GCCTCTCTCTCAGACGTTAGT

67 **Secondary Antibody Table (Table 3)**

Reagent name	Company	Catalog number
HRP-conjugated Rabbit Anti-Goat IgG H&L	Abcam	#ab6741
Anti-rabbit IgG, HRP-linked Antibody	Cell Signaling Technology	#7074
HRP-conjugated Goat Anti-Mouse IgG(H+L)	Proteintech	#SA00001-1

68 **Primary Antibody Table (Table 4)**

Reagent name	Company	Catalog number
DGAT2 Goat mAb	Abcam	#ab59493
DGAT2 Mouse mAb	Santa Cruz Biotechnology	#sc-293211
Spry1 Rabbit mAb	Cell Signaling Technology	#13013

β-Actin Rabbit mAb	Cell Signaling Technology	#4970
Anti-FLAG	Sigma-Aldrich	#F3165
HA-tag Rat mAb	Cell Signaling Technology	#7C9
JNK Mouse mAb	Santa Cruz Biotechnology	#sc-7345
Phospho-SAPK/JNK (Thr183/Tyr185) (81E11) Rabbit mAb	Cell Signaling Technology	#4668S
p44/42 MAPK (Erk1/2) (137F5) Rabbit mAb	Cell Signaling Technology	#4695S
Phospho-p44/42 MAPK (Erk1/2) (Thr202/Tyr204) Rabbit mAb	Cell Signaling Technology	#4370S
p38 MAPK Polyclonal antibody	Proteintech	#14064-1-AP
Phospho-p38 MAPK (Thr180/Tyr182) Rabbit mAb	Cell Signaling Technology	#4511S
NF-κB p65 Rabbit mAb	Cell Signaling Technology	#8242T
Phospho-NF-κB p65 (Ser536) Rabbit mAb	Cell Signaling Technology	#3033S
IκBα Mouse mAb (Amino-terminal Antigen)	Cell Signaling Technology	#4814T

Phospho-IκBα (Ser32) Rabbit mAb	Cell Signaling Technology	#2859T

RNA Primers Table (Table 5)

Gene Name	Primer Type	Sequence (5' → 3')
Il1b	Forward Primer	GAAATGCCACCTTTGACAGTG
	Reverse Primer	TGGAATCCCTCACACTCAGGACAG
Il6	Forward Primer	CTGCAAGAGACTTCCATCCAG
	Reverse Primer	AGTGTGATGAGAGCAGGGTCTGTGG
Il17a	Forward Primer	TCACGGTGTCCTCACACATCAG
	Reverse Primer	CGCCAAGGGAGTTAAAGACTT
Il22	Forward Primer	ATGAGTTTTTCCCTTATGGGAC
	Reverse Primer	GCTCGAAGGAGCAAACCTCAA
Il23	Forward Primer	AATAATGTGCCCGCATACCATG
	Reverse Primer	GCTCCCTTTGGAAGATGCTAG
Tnfα	Forward Primer	CTGAACTTCGGGGTGATCGG
	Reverse Primer	GGCTGTCCCTCTGATGGCATTTCAGA
Spry1	Forward Primer	ACACCTGCATGGTGGTGTTCA

	Reverse Primer	CTGCTATTCACATTGCTGGGTAT
Dgat2	Forward Primer	GGCGTACTCAGCGACTATCTT
	Reverse Primer	GGGCCTTATGCAGGAAACT
