# Supplementary material for: DGAT2 reduction and lipid dysregulation drive psoriasis development in keratinocyte-specific SPRY1-deficient mice
Source: JCI Insight. 2025 Jul 22;10(17):e192507. doi: 10.1172/jci.insight.192507 (PMC12487672; doi:10.1172/jci.insight.192507)

Full unedited blot for Figure 1

Used in the Figures

Full unedited blot for Figure 1G

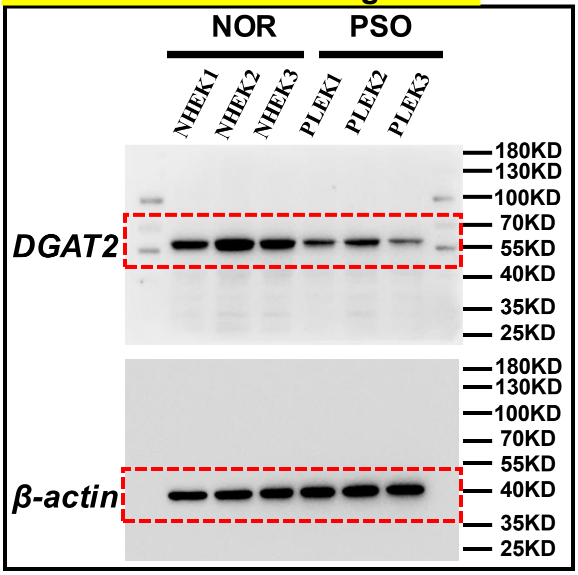

Full unedited blot for Figure 1H

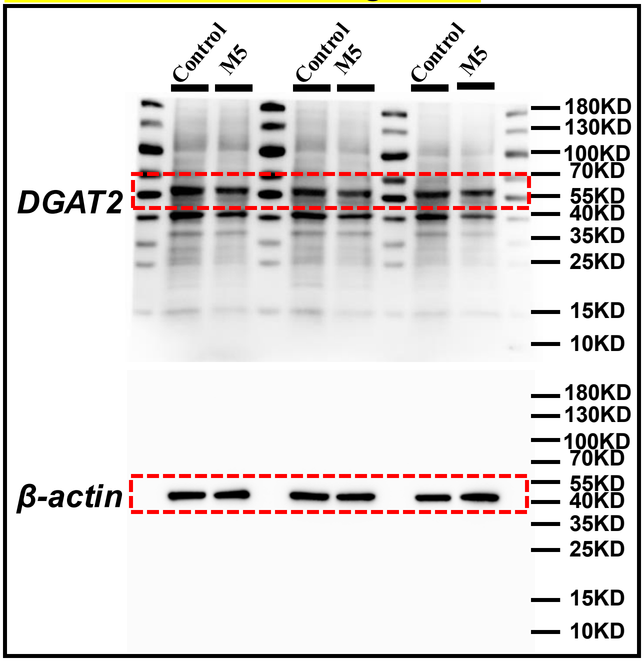

Merged with protein ladders

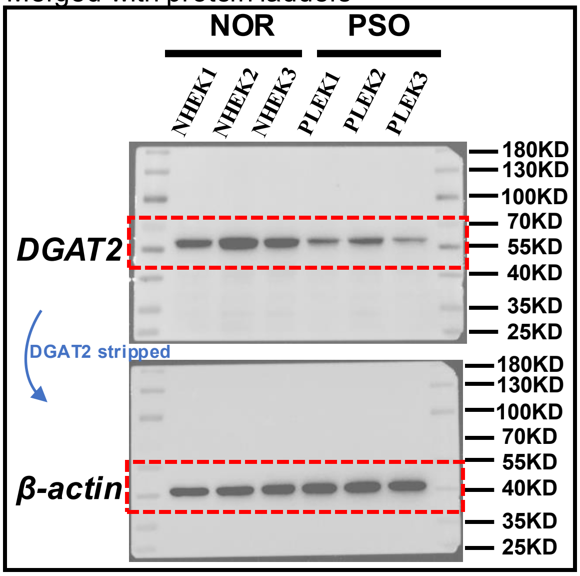

Merged with protein ladders

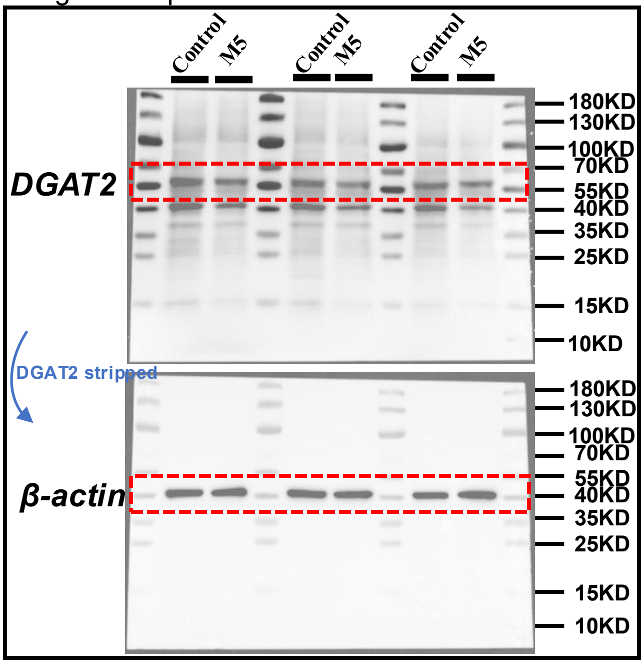

Full unedited blot for Figure 1I

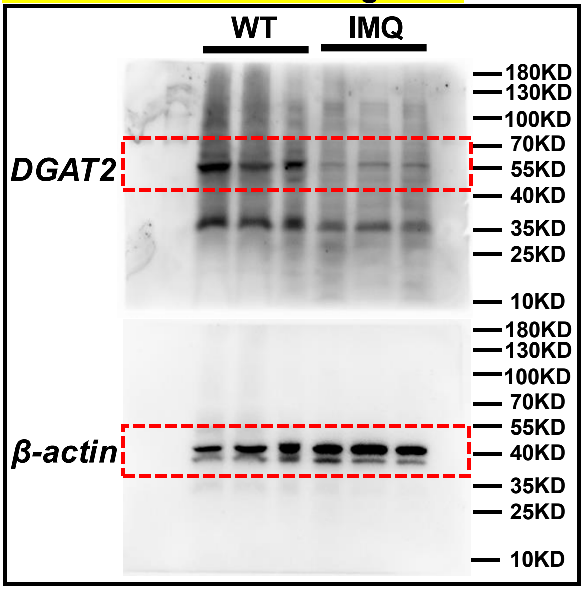

Merged with protein ladders

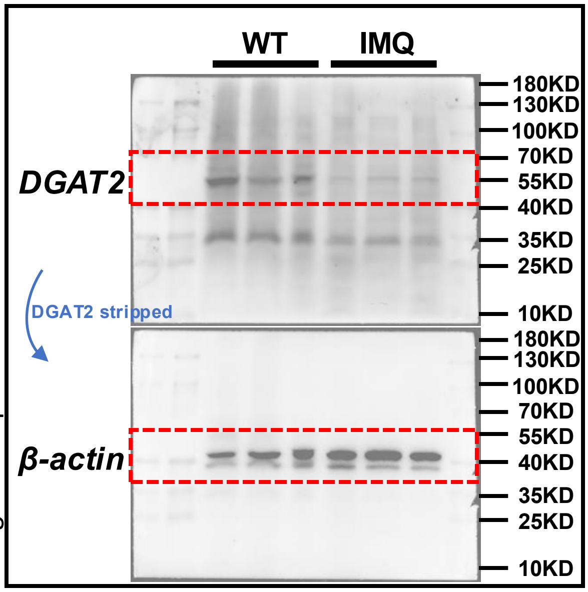

Full unedited blot for Figure 3

Used in the Figures

Full unedited blot for Figure 3E

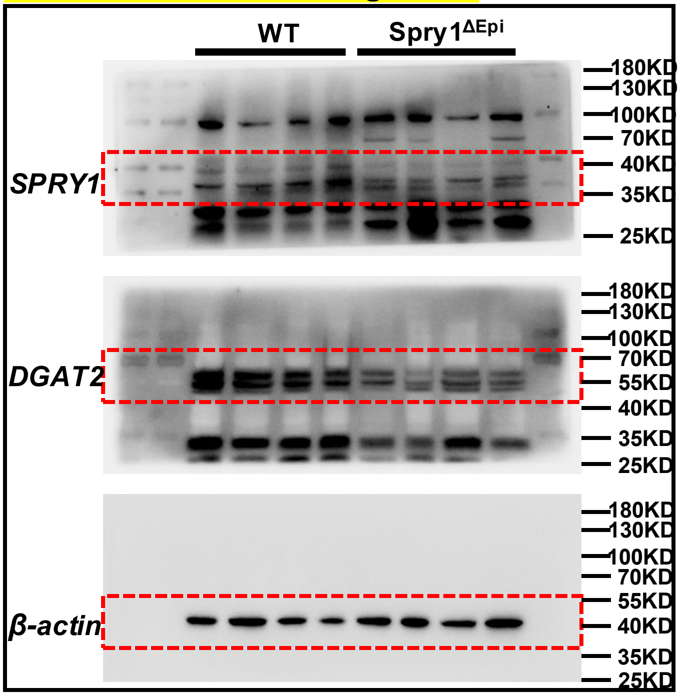

Merged with protein ladders

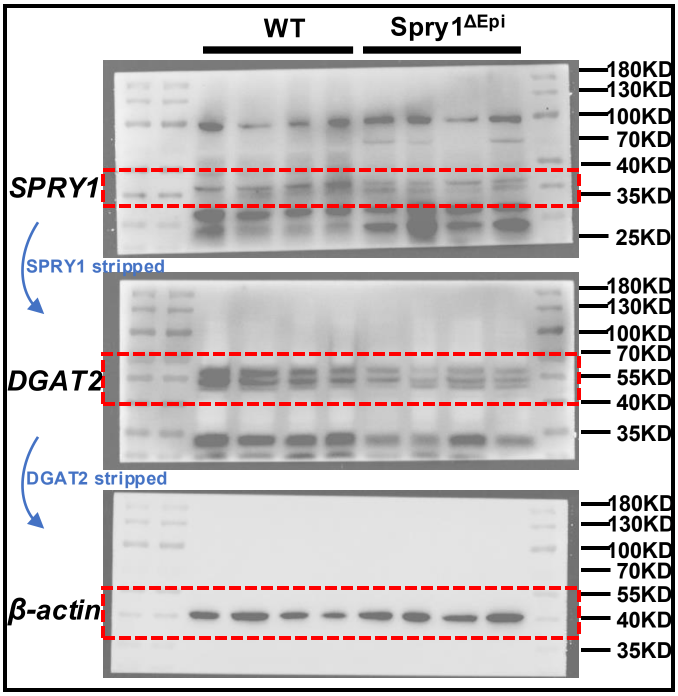

Full unedited blot for Figure 3F

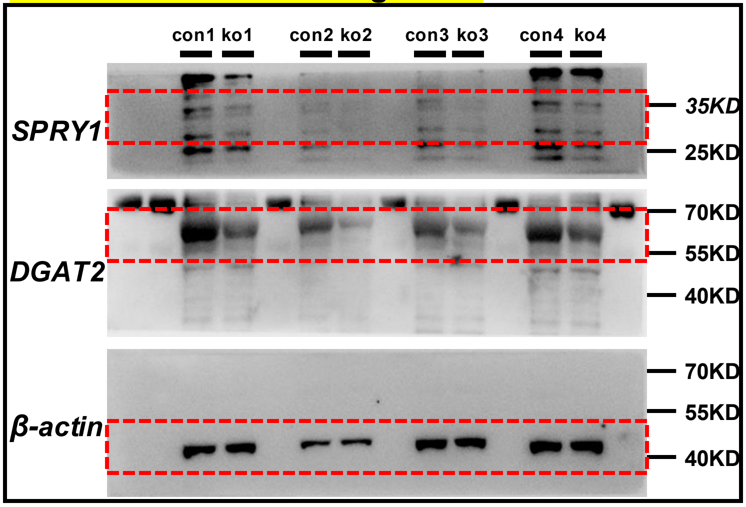

Merged with protein ladders

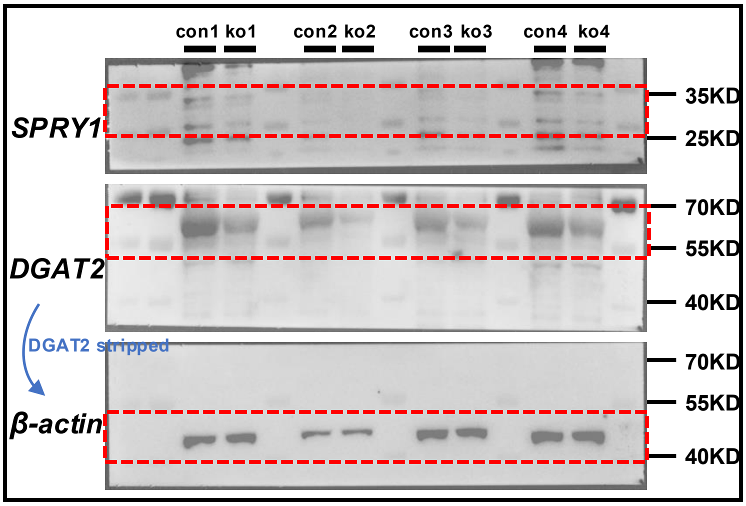

Full unedited blot for Figure 3G

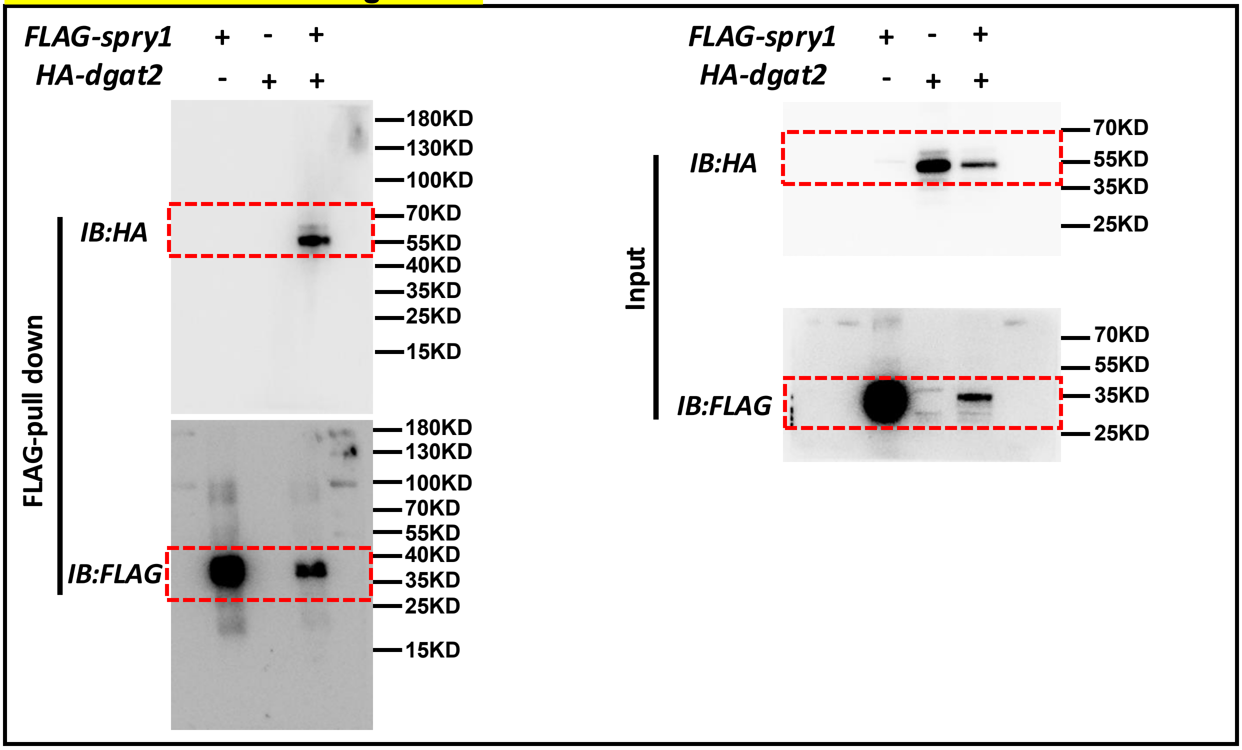

Merged with protein ladders

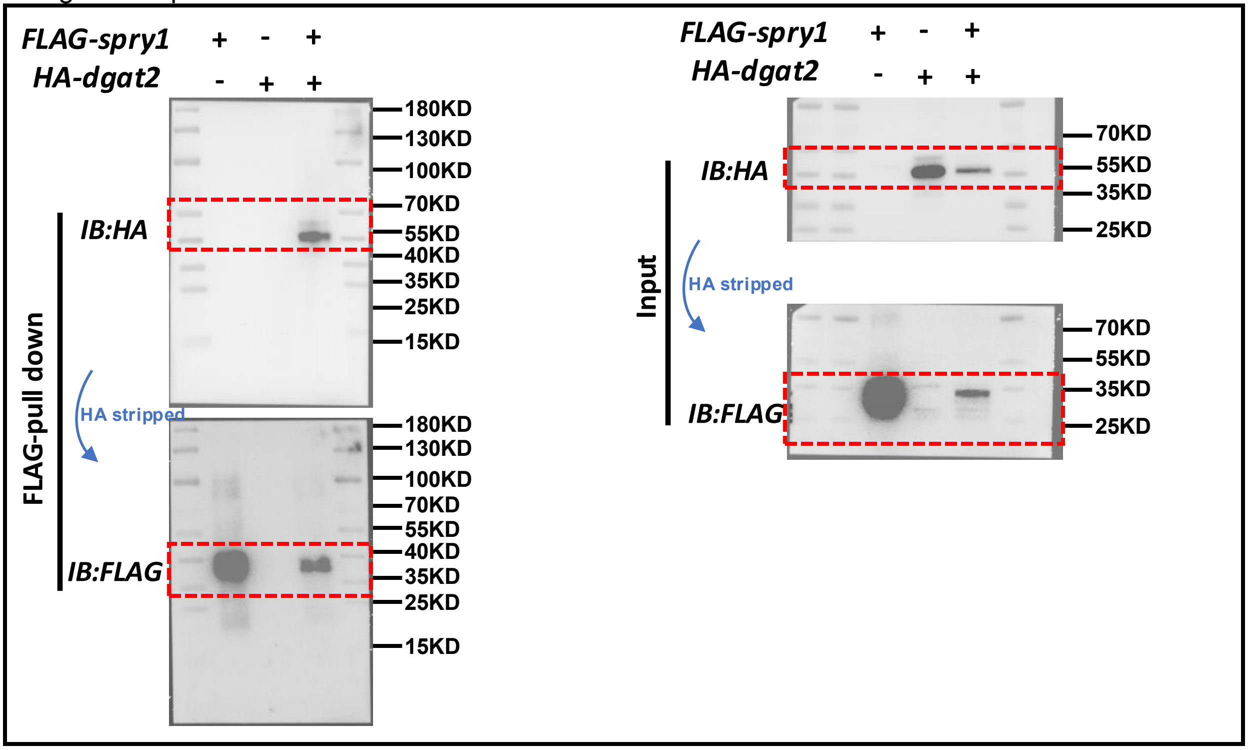

Full unedited blot for Figure 3H

Merged with protein ladders

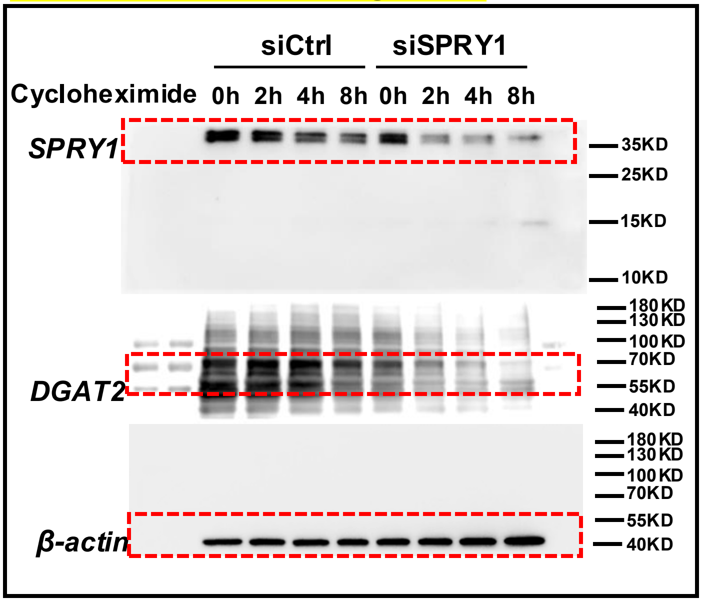

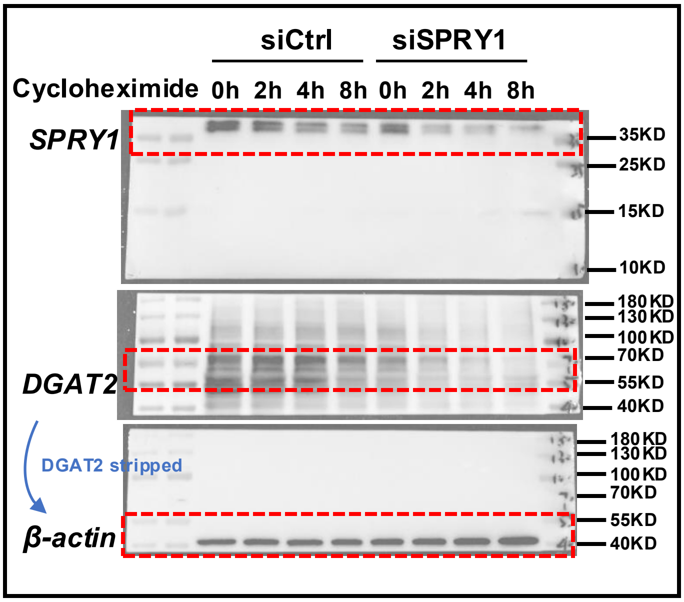

Full unedited blot for Figure 5

Used in the Figures

Full unedited blot for Figure 5G

Merged with protein ladders

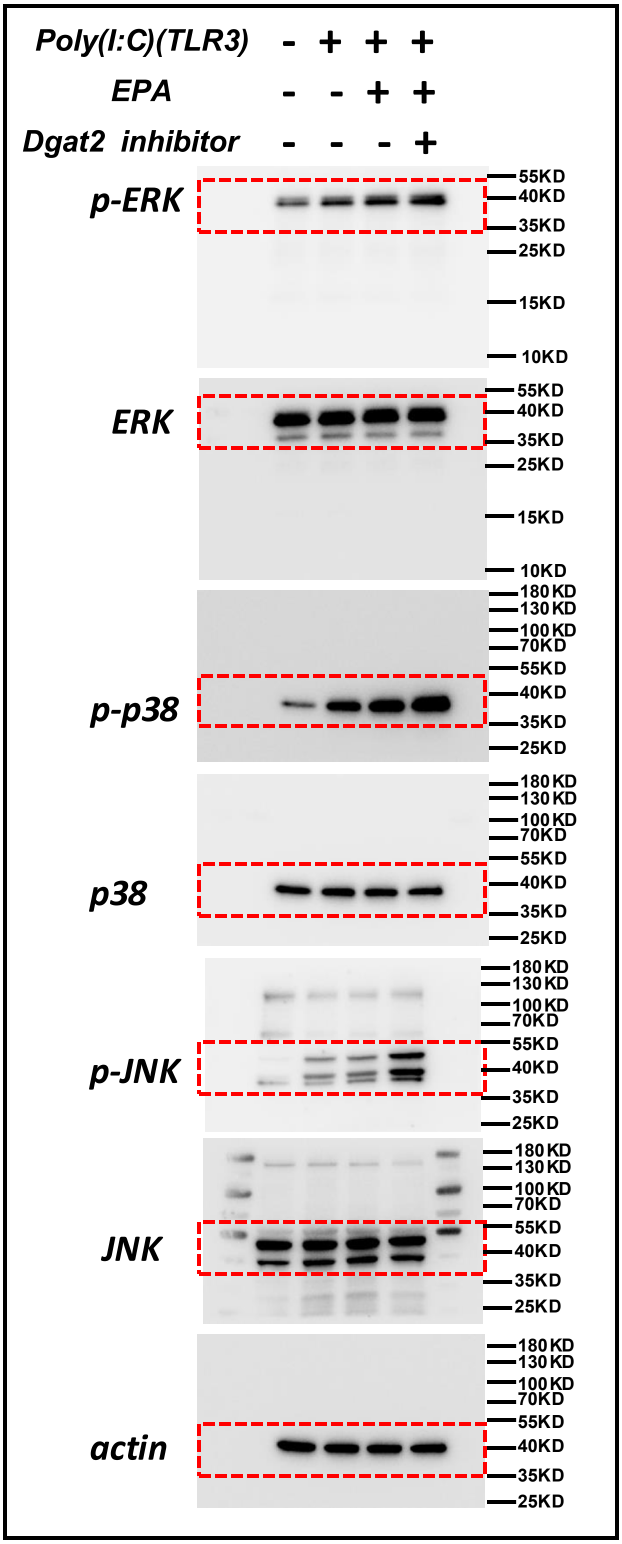

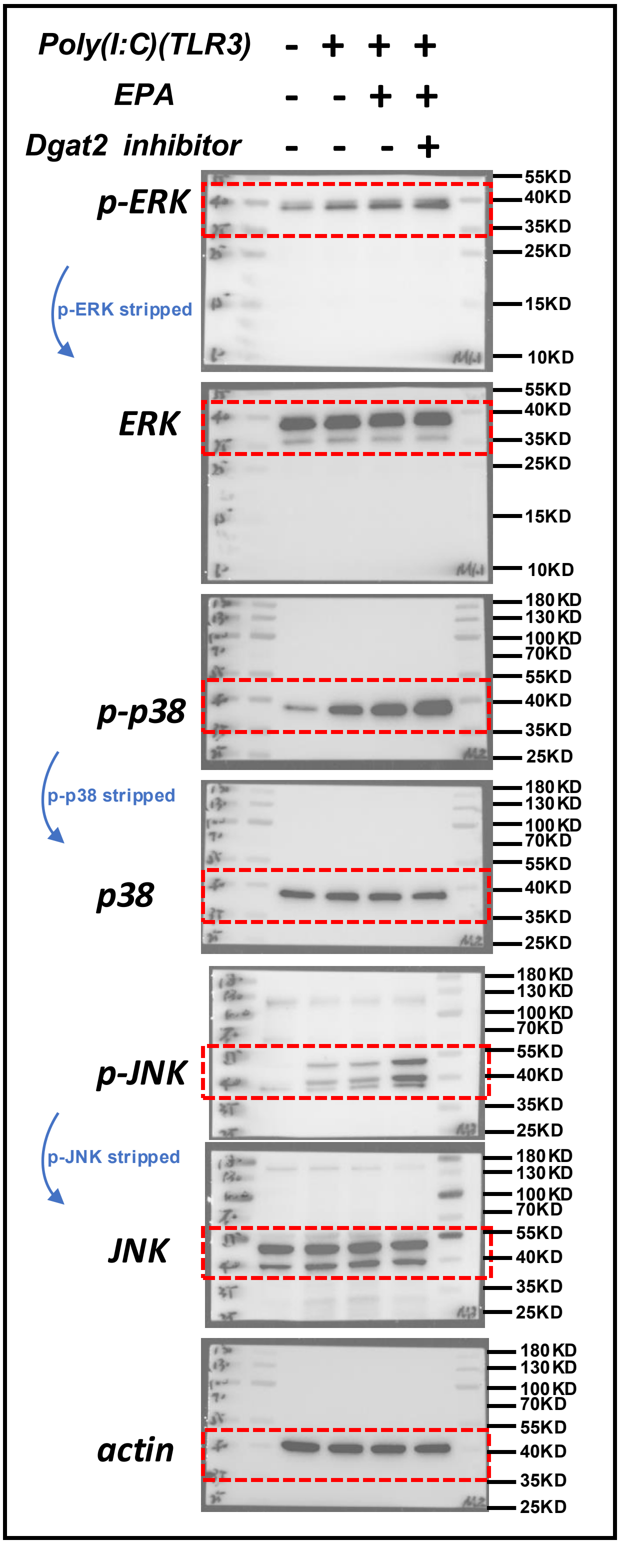

Full unedited blot for Figure 5H

Merged with protein ladders

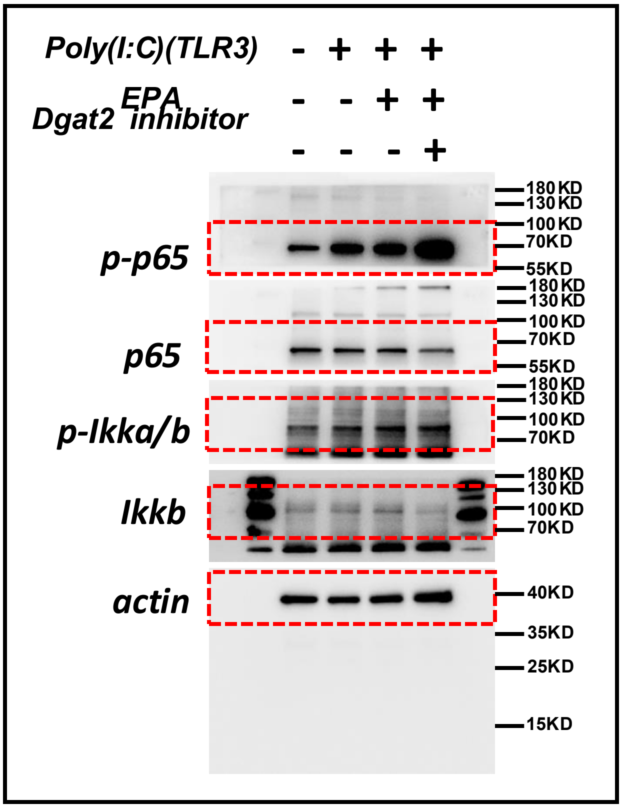

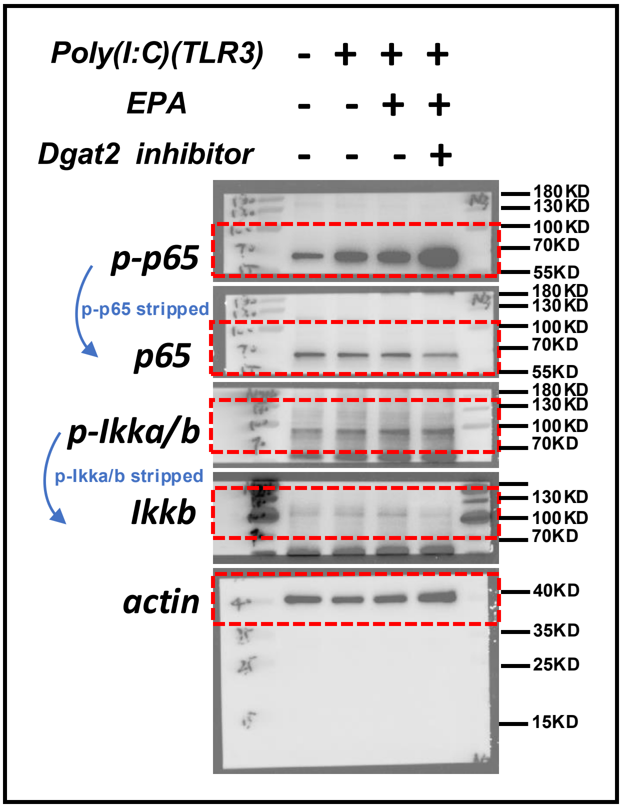

Full unedited blot for Figure 6

Used in the Figures

Full unedited blot for Figure 6A

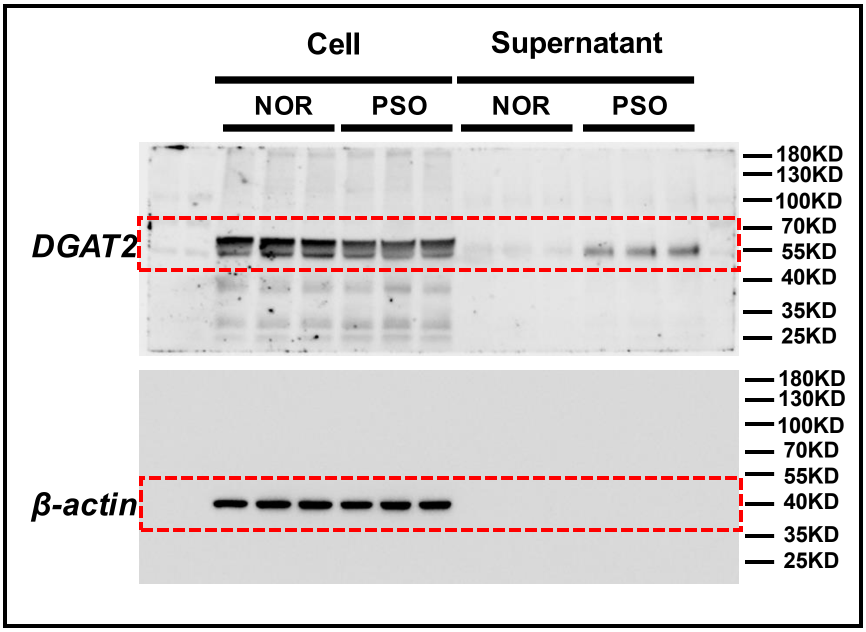

Merged with protein ladders

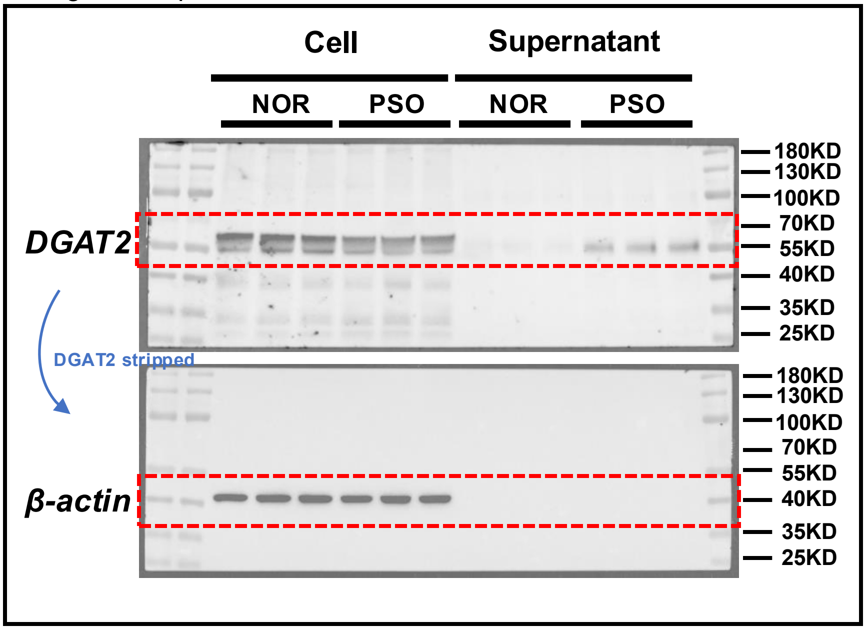

Full unedited blot for Supplemental Figure 3

Used in the Figures

Full unedited blot for Figure S3B

Merged with protein ladders

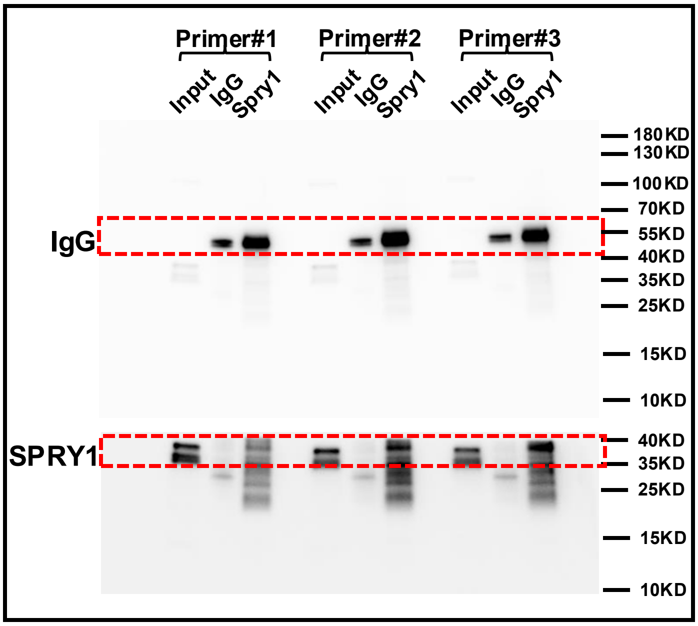

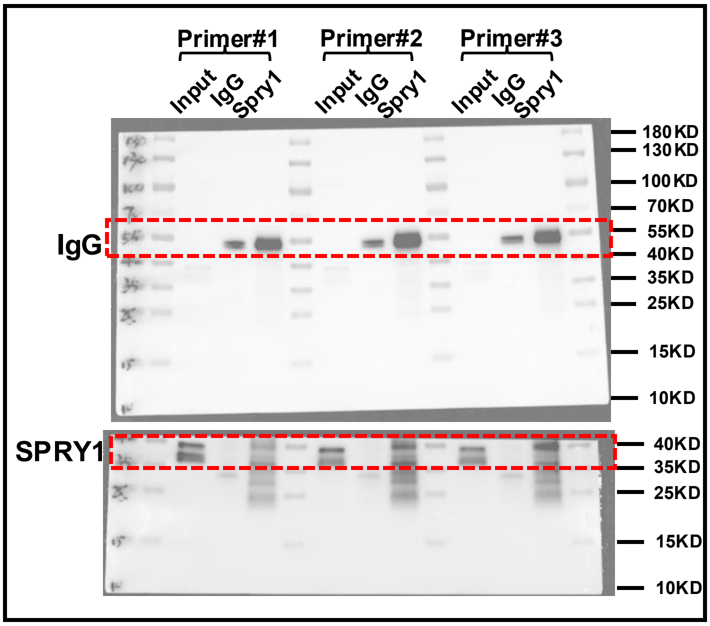

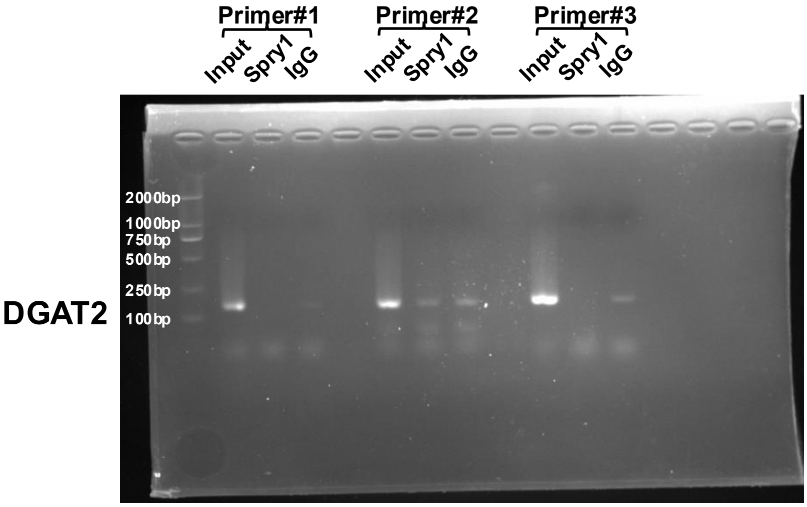

Full unedited blot for Figure S3C

Merged with protein ladders

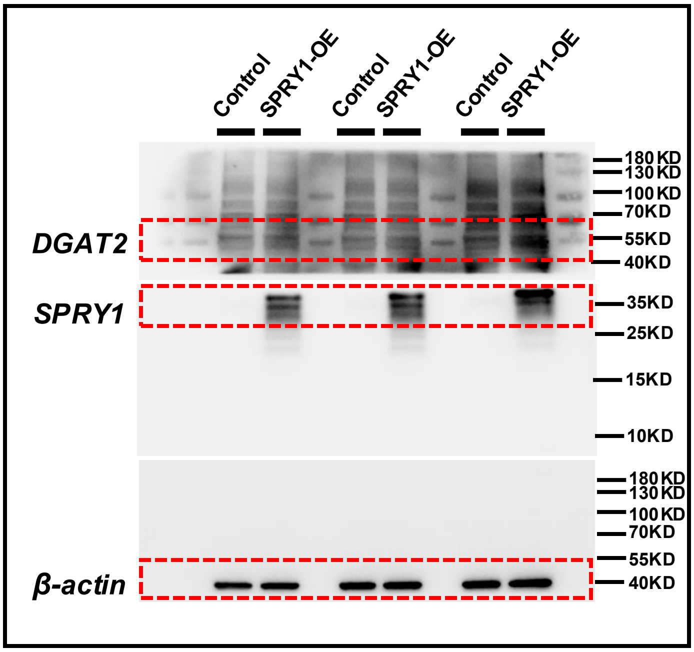

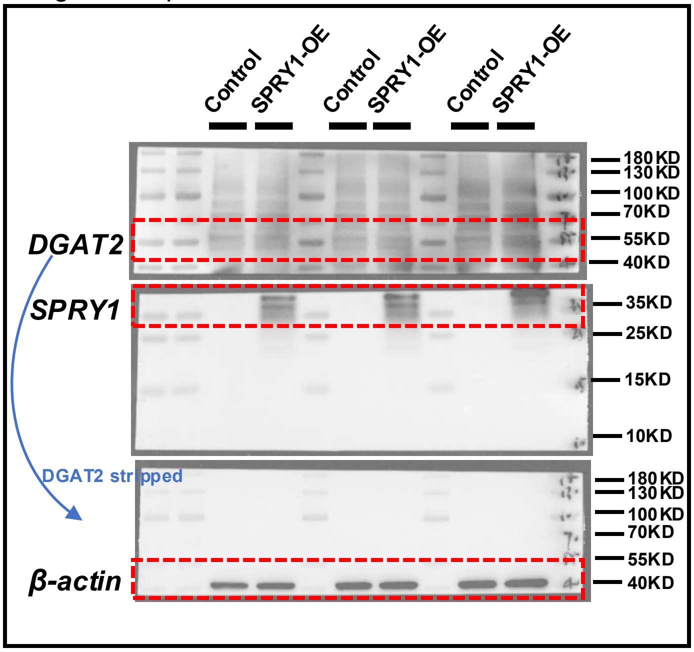

Supplement: Unedited blot and gel images [file jciinsight-10-192507-s059.pdf]
